# Chromosome-length genome assemblies of six legume species provide insights into genome organization, evolution, and agronomic traits for crop improvement

**DOI:** 10.1016/j.jare.2021.10.009

**Published:** 2021-11-03

**Authors:** Vanika Garg, Olga Dudchenko, Jinpeng Wang, Aamir W. Khan, Saurabh Gupta, Parwinder Kaur, Kai Han, Rachit K. Saxena, Sandip M. Kale, Melanie Pham, Jigao Yu, Annapurna Chitikineni, Zhikang Zhang, Guangyi Fan, Christopher Lui, Vinodkumar Valluri, Fanbo Meng, Aditi Bhandari, Xiaochuan Liu, Tao Yang, Hua Chen, Babu Valliyodan, Manish Roorkiwal, Chengcheng Shi, Hong Bin Yang, Neva C. Durand, Manish K. Pandey, Guowei Li, Rutwik Barmukh, Xingjun Wang, Xiaoping Chen, Hon-Ming Lam, Huifang Jiang, Xuxiao Zong, Xuanqiang Liang, Xin Liu, Boshou Liao, Baozhu Guo, Scott Jackson, Henry T. Nguyen, Weijian Zhuang, Wan Shubo, Xiyin Wang, Erez Lieberman Aiden, Jeffrey L. Bennetzen, Rajeev K. Varshney

**Affiliations:** a Center of Excellence in Genomics and Systems Biology, International Crops Research Institute for the Semi-Arid Tropics (ICRISAT), Hyderabad, India; b The Center for Genome Architecture, Department of Molecular and Human Genetics, Baylor College of Medicine, Houston, TX, USA; c Center for Theoretical Biological Physics, Rice University, Houston, TX, USA; d School of Life Sciences, North China University of Science and Technology, Tangshan, China; e Max Planck Institute of Molecular Plant Physiology, Potsdam, Germany; f UWA School of Agriculture and Environment, University of Western Australia, Perth, Australia; g BGI-Qingdao, BGI-Shenzhen, Qingdao 266555, China; h The Leibniz Institute of Plant Genetics and Crop Plant Research (IPK), Gatersleben, Germany; i BGI-Shenzhen, Shenzhen 518083, China; j National Key Facility for Crop Gene Resources and Genetic Improvement/Institute of Crop Sciences, Chinese Academy of Agricultural Sciences, Beijing 100081, China; k Institute of Oil Crops, Center of Legume Oil Crop Genetics and Systems Biology, Fujian Agriculture and Forestry University, Fuzhou, China; l Department of Agriculture and Environmental Sciences, Lincoln University, Jefferson City, MO, USA; m Crops Research Institute, Guangdong Academy of Agricultural Sciences, Guangzhou, China; n Shandong Peanut Research Institute, Shandong Academy of Agricultural Sciences, Qingdao, China; o Biotechnology Research Center, Shandong Provincial Key Laboratory of Crop Genetic Improvement, Ecology and Physiology, Shandong Academy of Agricultural Sciences, Ji’nan, China; p Center of Soybean Research of the State Key Laboratory of Agrobiotechnology and School of Life Sciences, The Chinese University of Hong Kong, Hong Kong; q Key Laboratory of Biology and Genetic Improvement of Oil Crops, Ministry of Agriculture and Rural Affairs, Oil Crops Research Institute of Chinese Academy of Agricultural Sciences, Wuhan, China; r China National GeneBank, BGI-Shenzhen, Shenzhen 518120, China; s Crop Genetics and Breeding Research Unit, USDA-ARS, Tifton, GA, USA; t Bayer Crop Sciences, Chesterfield, MO, USA; u Division of Plant Sciences and National Center for Soybean Biotechnology, University of Missouri, Columbia, MO, USA; v Shanghai Institute for Advanced Immunochemical Studies, ShanghaiTech, Pudong, China; w Broad Institute of MIT and Harvard, Cambridge, MA, USA; x Department of Genetics, University of Georgia, Athens, GA, USA; y State Agricultural Biotechnology Centre, Centre for Crop and Food Innovation, Food Futures Institute, Murdoch University, Murdoch, Western Australia, Australia; z The UWA Institute of Agriculture, University of Western Australia, Perth, Australia

**Keywords:** Evolution, Plant genomes, GWAS, Nodulation, QTL, Legumes

## Abstract

**Introduction:**

Legume crops are an important source of protein and oil for human health and in fixing atmospheric N_2_ for soil enrichment. With an objective to accelerate much-needed genetic analyses and breeding applications, draft genome assemblies were generated in several legume crops; many of them are not high quality because they are mainly based on short reads. However, the superior quality of genome assembly is crucial for a detailed understanding of genomic architecture, genome evolution, and crop improvement.

**Objectives:**

Present study was undertaken with an objective of developing improved chromosome-length genome assemblies in six different legumes followed by their systematic investigation to unravel different aspects of genome organization and legume evolution.

**Methods:**

We employed *in situ* Hi-C data to improve the existing draft genomes and performed different evolutionary and comparative analyses using improved genome assemblies.

**Results:**

We have developed chromosome-length genome assemblies in chickpea, pigeonpea, soybean, subterranean clover, and two wild progenitor species of cultivated groundnut (*A. duranensis* and *A. ipaensis*). A comprehensive comparative analysis of these genome assemblies offered improved insights into various evolutionary events that shaped the present-day legume species. We highlighted the expansion of gene families contributing to unique traits such as nodulation in legumes, gravitropism in groundnut, and oil biosynthesis in oilseed legume crops such as groundnut and soybean. As examples, we have demonstrated the utility of improved genome assemblies for enhancing the resolution of “*QTL-hotspot*” identification for drought tolerance in chickpea and marker-trait associations for agronomic traits in pigeonpea through genome-wide association study. Genomic resources developed in this study are publicly available through an online repository, ‘Legumepedia’.

**Conclusion:**

This study reports chromosome-length genome assemblies of six legume species and demonstrates the utility of these assemblies in crop improvement. The genomic resources developed here will have significant role in accelerating genetic improvement applications of legume crops.

## Highlights

• The study presents chromosome-length genome assemblies of six legume species.
• Evolutionary events that shaped the present day legumes are inferred.
• Expansion of gene families contributing to unique traits in legumes is explored.
• Demonstrated the utility of improved assemblies as better references.

## Graphical abstract

**Figure:**
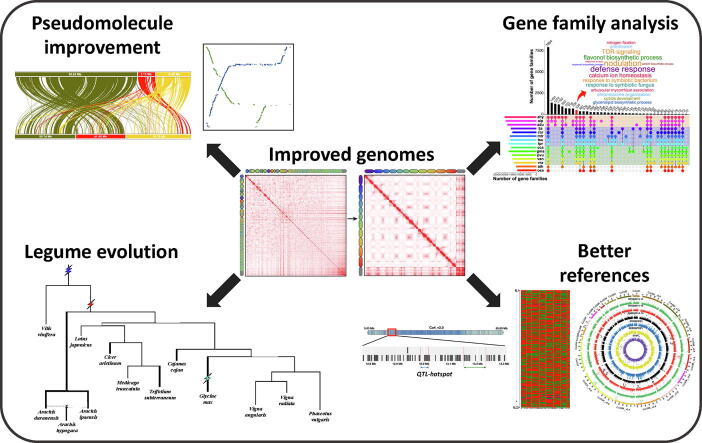

## Introduction

With 750 genera and 19,000 species, Leguminosae is the third largest family of angiosperms. The subfamily Papilionoideae is the largest of the three subfamilies (Caesalpinioideae, Mimosoideae, and Papilionoideae) and contains species of immense economic value globally as grain and forage legume crops. Being important commodities that provide protein for human consumption and fix atmospheric N_2_ for soil health, legume crops are indispensable for global food and nutritional security and environmental sustainability [1]. The majority of these legume crops are grown by smallholder farmers in the developing world under a range of severe biotic and abiotic stresses. As a result, worldwide crop productivity for legumes is very low [1]. Very recently, the 5Gs approach for crop improvement has been suggested [2]. The 1st G stands for *G*enome assembly, providing opportunities to develop genomic resources that can be used in breeding programs as well as for understanding genome structure and evolution.

Due to advances in next-generation sequencing (NGS) technologies, genome assemblies, though fragmented, have been developed for several legume crops. For instance, N50 (scaffolds) values were 39.99 Mb, 0.51 Mb, 50.39 Mb, and 0.28 Mb in chickpea (*Cicer arietinum*, CaGA v1.0 [3]), pigeonpea (*Cajanus cajan*, C.cajan_V1.0 [4]), soybean (*Glycine max*, glyma Lee gnm1 [5]), and subterranean clover (*Trifolium subterraneum*, TSUd_r1.1 [6]), respectively. In addition, genome assemblies for two diploid progenitors of cultivated groundnut (*Arachis hypogaea*) - *Arachis ipaensis* (Araip1.1, N50 = 136.18 Mb), *Arachis duranensis* (Aradu1.1, N50 = 110.04 Mb) - were also developed mainly based on short sequence reads [7].

Owing to high levels of heterozygosity, polyploidy, and extensive repeat content, assembling plant genomes has been a challenge. The traditional ways of anchoring contigs and scaffolds can lead to erroneous genome assemblies that err in order and/or orientation [8]. Over the past five years, considerable efforts have been focused on improving the genome assemblies, including the development of near-finished genomes using long-read sequencing technologies. In recent years, high-throughput chromosome conformation capture sequencing (Hi-C) analysis has gained popularity in terms of improving the existing draft genome assemblies and in yielding chromosome-length scaffolds by using the concept of chromatin contact frequency [9], [10], [11].

Here, we used *in-situ* Hi-C data to develop improved chromosome-length genome assemblies of chickpea (referred to as Cicar.CDCFrontier_v2.0), pigeonpea (Cajca.Asha_v2.0), soybean (Glyma.Lee_v2.0), wild groundnut relatives *A. duranensis* (Aradu.V14167_v2.0) and *A. ipaensis* (Araip.K30076_v2.0), and subterranean clover (Trisu.Daliak_v2.0). We used these chromosome-length genome assemblies to evaluate evolutionary divergence among legumes, re-date major evolutionary events, and describe massive gene loss and gain events. We performed comparative analyses across the Papilionoideae family to identify species-specific and expanded gene families. We have demonstrated the utility of these chromosome-length genome assemblies to enhance precision and resolution in the fine mapping of drought tolerance in chickpea and marker-trait associations for agronomic traits in pigeonpea. We have provided all these datasets through an online repository called “Legumepedia” (https://cegresources.icrisat.org/legumepedia/index.php).

## Materials and methods

### Generation of Hi-C data and development of chromosome-length (“C”) genome assemblies

*In situ* Hi-C was performed as described previously [12] using fresh leaves from chickpea cv. CDC Frontier, pigeonpea cv. Asha, soybean cv. Lee, *A. duranensis* V14167, *A. ipaensis* K30076 and subterranean clover cv. Daliak. Before harvesting, mature, dry seeds were grown for 2–3 weeks in sterilized potting mix and dark treated for 2–3 days. We constructed one *in situ* library each for chickpea, subterranean clover and *A. ipaensis*; two *in situ* libraries each for pigeonpea, soybean, and *A. duranensis* (**Table S1**). These libraries were sequenced using the HiSeq X Ten and NextSeq 500 instruments. The generated Hi-C reads were used to anchor, order, orient, and correct misjoins in the existing draft genome assemblies (“D assemblies”) of six legumes (chickpea [3], pigeonpea [4], soybean [5], subterranean clover [6], *A. duranensis* [7], *A. ipaensis* [7]) using the 3D de novo assembly (3D-DNA) pipeline [10]. The resulting assemblies were then polished using the Juicebox Assembly Tools [13]. The resulting contact maps were visualized using Juicebox visualization software [14]. Further, the whole genome alignments between the “D” and respective “C” assemblies were performed and visualised using minimap2 v2.17 [15] and D-GENIES v1.2.0 [16], respectively.

### Identification of repeats

Both *de novo* and homology-based repeat identification approaches were used to identify and annotate repeats from the “C assemblies” of all six legumes. First, a *de novo* repeat library for each of the studied genomes was constructed using RepeatModeler version open-1.0.10 with default parameters [17]. The *de novo* repeat library, thus obtained, was combined with the known repeats from RepBase version 20170127 to generate a custom repeat library for each genome [18]. These libraries were then used to screen the assembled genomes for repeats using RepeatMasker version open-4.0.7 [19] (“-u -gff -e ncbi -xsmall”).

### Gene prediction and annotation

From each of the “C assemblies”, gene models were predicted by integrating homology-based prediction, *ab initio* prediction and transcriptome-based evidence. The non-redundant protein sequences of several species, including *Medicago truncatula*, common bean, mungbean, and soybean, as well as protein sequences from Swiss-Prot, were separately aligned to the “C assemblies” using BLAT v36 [20]. The matched hits were further processed with GeneWise [21] (Wise2.4.1 package). For *ab initio* prediction, publicly available RNA-Seq datasets for chickpea, pigeonpea, soybean, subterranean clover, *A. duranensis*, and *A. ipaensis* (**Table S2**) were aligned to the respective “C assemblies” using Hisat2 [22] (v2.1.0) with default parameters. These alignments were further used as input evidence for the BRAKER pipeline [23] (version 2.1.0). For transcriptome-based prediction, the transcriptomes assembled using Trinity v2.0.6 [24] were aligned to the respective “C assemblies” using the PASA pipeline [25] (v2.3.3) with both GMAP [26] (v2018-07–04) and BLAT. Gene model evidences obtained from the above three approaches were integrated by EVidenceModeler [27] (EVM; v1.1.1) into a non-redundant set of gene models.

Functional annotations were assigned to the genes using BLASTP (1E-05) according to the best hits to the NCBI non-redundant, Swiss-Prot, and TrEMBL databases [28]. Further, InterProScan [29] (v5.39–77.0; with default databases) was used to identify conserved domains and motifs in the proteins encoded by the predicted gene models. Gene Ontology IDs for each gene were obtained from the corresponding InterPro entry. For ribosomal RNA (rRNA) prediction, each genome was searched against rRNA sequences from *Arabidopsis* and rice using BLASTN (1E-05). The transfer RNA (tRNA) genes were identified using the tRNAscan-SE v2.0 [30]. Further, microRNA (miRNA) and small nucleolar (snoRNA) genes were predicted using a similarity search against the Rfam database [31] (release 14.2) using Infernal (v1.1.3) software [32]. The pseudogenes were predicted by integrating results from two different pipelines to retain the commonly predicted genes [33], [34].

### Gene family analysis

The predicted protein sequences from chickpea, pigeonpea, soybean, subterranean clover, *A. duranensis*, *A. ipaensis* together with *Medicago*, lotus, cultivated groundnut, mungbean, adzuki bean, common bean, red clover, *Arabidopsis*, and rice were analyzed using OrthoFinder v2.3.7 [35] to identify sets of orthologous genes. Single copy orthologs were used to construct species phylogenetic tree. Orthogroups obtained from OrthoFinder were further processed by CAFE v4.1 [36] to analyze gene family size changes. Further, the RGAugury pipeline [37] (version 2017–10-21) was used to identify resistance gene analogs (RGAs) from “C assemblies” of the studied legumes. The identified RGAs were then classified into different subfamilies based on the presence/absence of specific domains. The nodulation-related genes in the studied legumes were identified by performing reciprocal and bi-directional best hits searches (E-value threshold of 1E-05) against the predicted nodulation-related genes from previous studies [38], [39], [40]. The predicted nodulation genes were subjected to phylogenetic analysis using the Neighbor-joining method implemented in MEGA X (https://www.megasoftware.net/) with 1,000 bootstraps. To detect known transcription factors (TFs) in the genomes of the six studied legumes, we used the Plant Transcription Factor Database [41] (PlantTFDB version 5.0).

### Inferring gene colinearity and genomic homology

Chromosomes from within a genome or different genomes were compared using the predicted gene models for all the legumes described earlier [42]. In brief, the protein sequences were aligned against each other to identify potentially homologous genes using BLASTP (1E-05). The homologous gene pairs thus identified were represented as dot plots using the Perl graphics drawing module GD. In these dot plots, homologous gene pairs were shown in red, blue, and gray to denote the best, secondary, and other matches, respectively, to help distinguish homologies related to different events. Subsequently, these homologous genes were used as an input for identification of colinear genes using ColinearScan [43] (maximum gap of 50 intervening genes). Large gene families (30 or more copies in the genome) were not considered for this analysis. Further, the reference genome of grape (*Vitis vinifera*) [44] was used as an outgroup species for deciphering the chromosomal homology across the studied legumes as the genome structure of grape remained conserved before and after the eudicot-common hexaploid (ECH) event. The grape genome was highly significant in distinguishing the nature of the origin of the paralogous blocks within legumes (whether the paralogous blocks were the result of ECH or some other event).

### Ks estimation and evolutionary dating

Synonymous nucleotide substitutions on synonymous sites (Ks) were estimated using the Nei-Gojobori approach [45] implemented by using the BioPerl Statistical module. The homologous genes related to different polyploidization events were inferred using the intra- and inter-genomic homologous gene dot plots. For homologous blocks within a genome or between genomes, the median Ks values were calculated. Since the Ks values of the gene set of different evolutionary events are different, the median Ks values helped distinguish homologous blocks produced by different events. For instance, the smaller Ks values indicate less diverged homologous genes and a recent evolutionary event (refer Wang et al. [46] for detailed methodology). The key evolutionary events were dated using the genomics dating approach described previously [42]. In brief, the steps included, i) dissecting Ks distribution into several normal distributions, ii) using the first component of the normal distribution to define the location of the Ks distribution, iii) aligning the Ks normal distributions of homologs from different genomes but produced by the same event to correct evolutionary rates.

### High-resolution mapping of “*QTL-hotspot*” in chickpea

A RIL population (RIL3) developed by crossing ICC 4958 (a drought-tolerant genotype) and ICC 1882 (a drought-sensitive genotype) was used for linkage mapping [47]. The skim sequencing data generated in Kale et al. [48] was used for variant calling. The whole-genome sequencing (WGS) data of the RIL population, along with parental lines, were mapped against the new “C assembly”. The SNPs identified were then used to identify recombinantion breakpoints, which were then used as markers for the construction of a high-density bin map. Further, QTL analysis was also carried out using a high-density bin map and the phenotypic data for 17 drought-related traits and two drought indices. Subsequently, QTL analysis results were also used to redefine the earlier identified “*QTL-hotspot*” region. The methodology described in Kale et al. [48] was adopted for performing the above steps. The results from the current study were compared with those from Kale et al. [48] to assess the quality of genome assembly.

### Construction of genetic map in pigeonpea

An F_2_ mapping population derived by crossing ICPA 2039 × ICPL 87119 was used for high-density linkage map construction in the present study. A total of 336 progenies, along with parental lines, were genotyped using the “Axiom *Cajanus* SNP Array”, which resulted in the identification of 11,697 polymorphic markers [49]. The R/qtl package [50] from the R environment was used for linkage map construction. Initially, the highly distorted markers were removed (*P*-value < 1E-04). The recombination frequencies were calculated using est.rf function, while grouping was done using formLinkageGroups function. Finally, the marker distance was estimated using the kosambi mapping function [51]. The markers were mapped on both “D” and “C” assemblies, and genetic map and genome assembly comparison was carried out to assess the assembly quality.

### Variant calling and genome-wide association study (GWAS) in pigeonpea

The WGS data of pigeonpea [52] was retrieved from NCBI (BioProject ID: PRJNA383013). The raw reads were subjected to filtering using Trimmomatic v0.39 [53] to obtain a set of clean reads. The clean reads were then mapped on both “D” and “C” assemblies of pigeonpea using BWA-mem v0.7.17 [54]. Resulting alignment files were processed to remove PCR duplicates using PicardTools v2.20.2 and subjected to variant calling using HaplotypeCaller and GenotypeGVCFs of Genome Analysis Toolkit v4.1.2.0 [55] (GATK). The obtained SNPs were filtered using GATK filters (QD < 2.0 || FS > 60.0 || MQ < 40.0 || MQRankSum < -12.5 || ReadPosRankSum < -8.0). Identified SNPs were studied for their effects using SnpEff release 4.3t [56]. Further, identified SNPs, along with phenotyping data collected from Varshney et al. [52] and Zhao et al. [57], were subjected to GWAS analysis using the FarmCPU method [58]. The phenotyping data for nine agronomic traits (days to 50% flowering (DF), days to 75% maturity (DM), primary branches per plant (PBPP), secondary branches per plant (SBPP), plant height (PH), pods per plant (PODSPP), seeds per pod (SEEDSPP), 100 seed weight (100SDW), and seed yield (SY)) was used in the study. The details of the phenotyping procedure have been described in Varshney et al. [52]. We performed variant calling and GWAS using both “D” and “C” assemblies of pigeonpea to avoid any methodological bias.

## Results

### Chromosome-length genome assemblies (“C assemblies”)

**Fig. 1:**
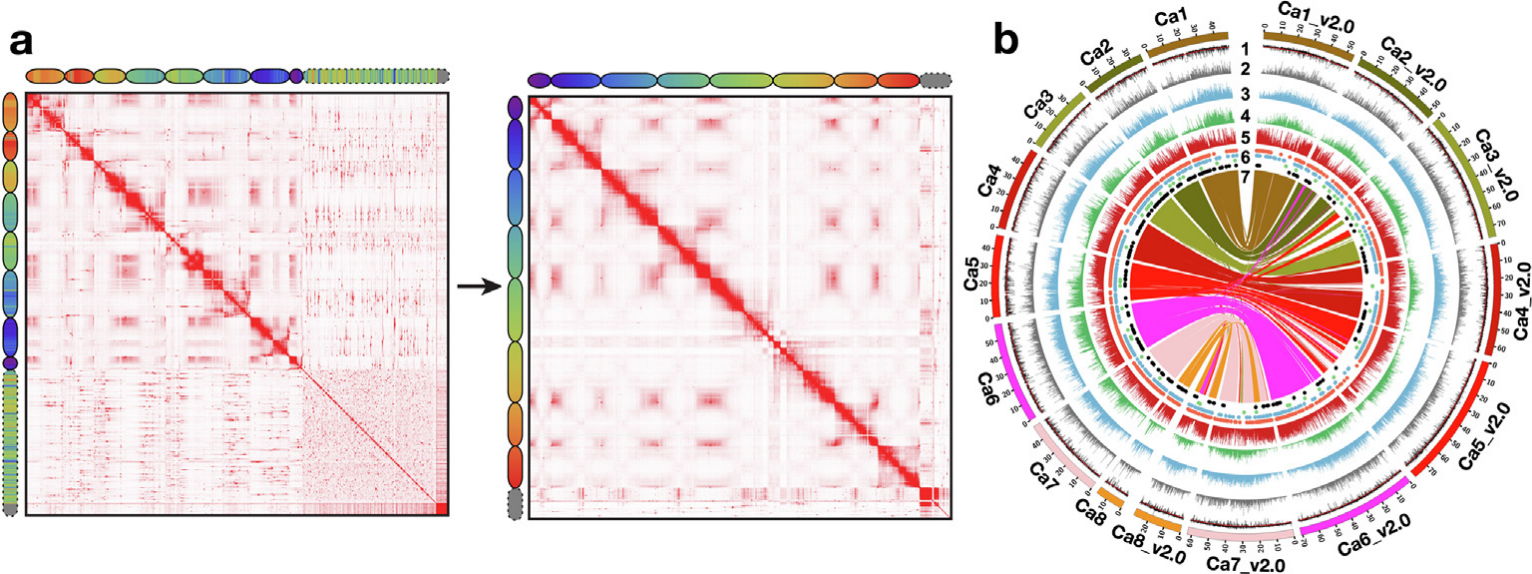
**Genomic landscape of “D” and Hi-C guided “C” assemblies of chickpea. (a)** Contact matrices generated by aligning the same Hi-C data to “D” (CaGA v1.0; left) and Hi-C guided “C” (Cicar.CDCFrontier_v2.0; right) assemblies. The color intensity in the matrices indicates the number of reads supporting co-localization of a pair of loci in the nucleus. Chromograms show the correspondence between loci in “D” and “C” assemblies. **(b)** A circos representing genomic features of both “D” and “C” assemblies. Different tracks in the circos represent (1) GC content density, (2) DNA repeat density, (3) LTR copia repeat density, (4) LTR gypsy repeat density, (5) gene density, (6) non-coding genes (transfer RNAs (in orange color); small nucleolar RNAs (in blue); ribosomal RNAs (in green); microRNAs (in black)), (7) synteny of “C” and “D” assemblies. Density bin size 20 kb. The links are colored based on the pseudomolecules of the “D assembly”. (For interpretation of the references to color in this figure legend, the reader is referred to the web version of this article.)

**Fig. 2:**
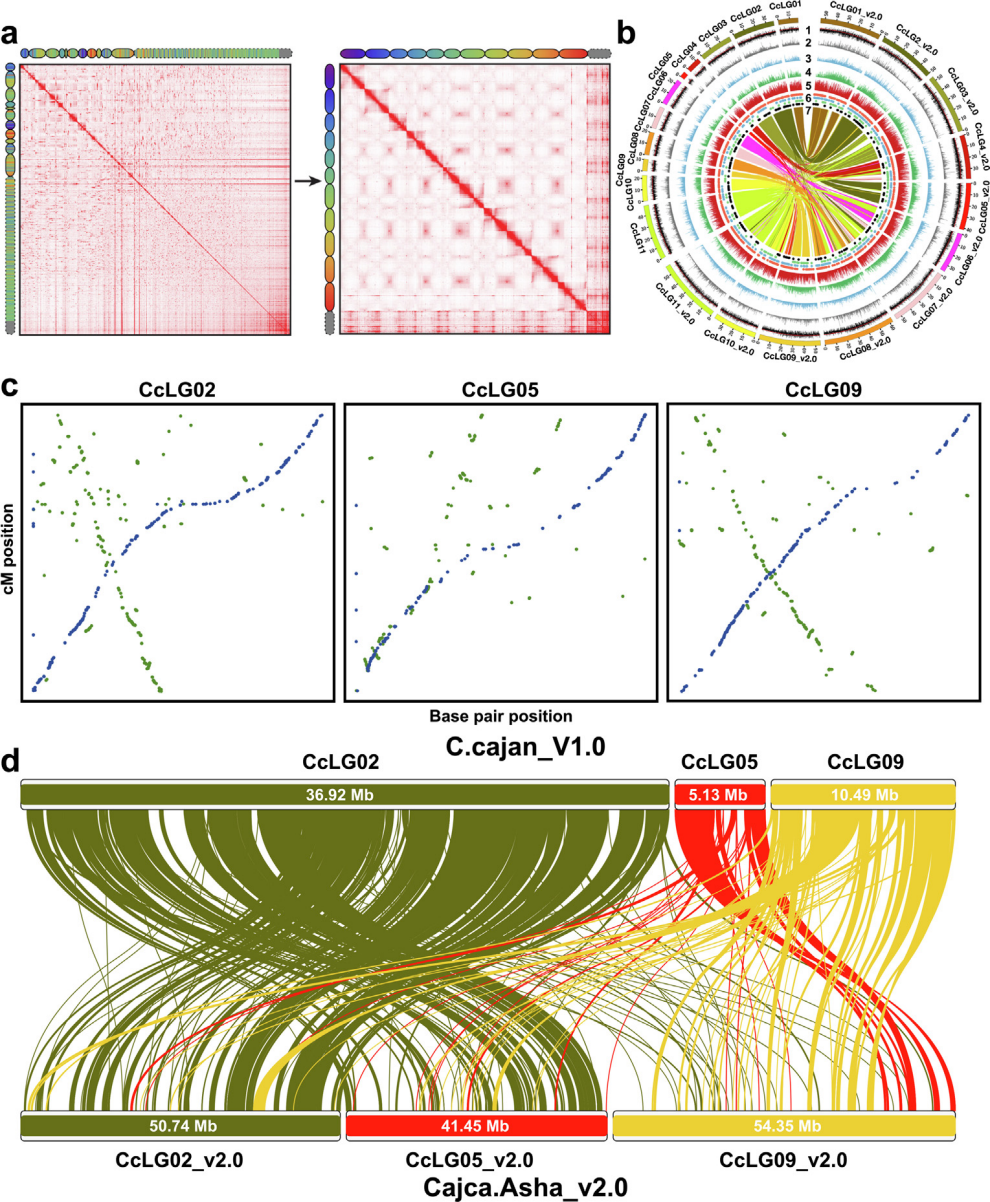
**Genomic landscape of “D” and Hi-C guided “C” assemblies of pigeonpea. (a)** Contact matrices generated by aligning the same Hi-C data to “D” (C.cajan_V1.0; left) and Hi-C guided “C” (Cajca.Asha_v2.0; right) assemblies. The color intensity in the matrices indicates the number of reads supporting co-localization of a pair of loci in the nucleus. Chromograms show the correspondence between loci in “D” and “C” assemblies. **(b)** A circos representing genomic features of both “D” and “C” assemblies. Different tracks in the circos represent (1) GC content density, (2) DNA repeat density, (3) LTR copia repeat density, (4) LTR gypsy repeat density, (5) gene density, (6) non-coding genes (orange, transfer RNAs; blue, small nucleolar RNAs; green, ribosomal RNAs; black, microRNAs), (7) synteny of “C” with “D” assemblies. The links are colored based on the pseudomolecules of “D assembly”. Density bin size 20 kb. **(c)** Genetic map and genome assembly comparison of three pseudomolecules in the “D” (green dots) and “C” (blue dots) assemblies. The x-axis and y-axis represent the coordinates of genome assembly and genetic map, respectively. **(d)** Synteny analysis of pseudomolecules CcLG02, CcLG05, and CcLG09 in “D assembly” with pseudomolecules CcLG02_v2.0, CcLG05_v2.0, and CcLG09_v2.0 in “C assembly”. (For interpretation of the references to color in this figure legend, the reader is referred to the web version of this article.)

To improve existing draft genome assemblies (“D assemblies”) [3], [4], [5], [6], [7], Hi-C sequence data ranging from 44.30X (*Arachis ipaensis*) to 106.34X (soybean) coverage were generated for six legume genomes (**Table S1**). The generated sequencing data in each species were used to anchor, order, orient, and correct misjoins in the “D assemblies”, thereby generating chromosome-length genome assemblies (“C assemblies”) (Fig. 1**a and 2a**; Figs. S1-S4). The “C assemblies” developed using Hi-C linking information resulted in scaffold N50 sizes ranging from 51.53 Mb (soybean) to 136.73 Mb (*A. ipaensis*) (Table 1). The repeat content in “C assemblies” was in the range of 41.14% (subterranean clover) to 69.98% (*A. ipaensis*). In agreement with the pattern in most other plant genomes [59], long-terminal repeat (LTR) retrotransposons were the most abundant class of repetitive DNA in all “C assemblies” (**Table S3**). The GC content in “C assemblies” of the studied legumes varied from 31.03% in chickpea to 36.84% in *A. ipaensis* (Table 1; Fig. S5).

**Table 1:** Statistics of genome assembly and annotation for six legume species.

Species	*C. arietinum*	*C. cajan*	*G. max*	*T. subterraneum*	*A. duranensis*	*A. ipaensis*
**Assembly features**						
*Total assembly size (in Mb)*	530.27	594.80	1015.36	473.15	1067.49	1349.51
*Total no of pseudomolecules*	8	11	20	8	10	10
*Number of scaffolds (>=10 kb; excluding pseudomolecules)*	717	839	73	143	946	474
*N50 (in Mb)*	65.80	53.90	51.53	56.31	109.37	136.73
*Assembly anchored in pseudomolecules*	92.59%	91.35%	99.33%	94.43%	96.98%	99.00%
*GC content*	31.03%	32.79%	34.93%	33.32%	35.81%	36.84%

**Protein-coding genes**						
*No of protein*-*coding genes*	25,105	29,482	51,839	37,474	33,810	38,783
*Mean gene length (in bp)*	4085	4437	4021	3666	3543	3447
*No of transcripts*	31,457	36,591	55,275	44,693	37,630	42,435
*Longest gene (in bp)*	80,819	97,397	97,943	96,273	96,307	93,505
*Shortest gene (in bp)*	157	150	150	155	152	154
*No of genes annotated*	24,489 (97.55%)	28,609 (97.04%)	51,088 (98.55%)	36,818 (98.25%)	32,456 (96.00%)	37,126 (95.73%)
*No of transcripts annotated*	30,819 (97.97%)	35,700 (97.56%)	54,520 (98.63%)	44,016 (98.49%)	36,236 (96.30%)	40,706 (95.93%)

**Non-coding genes**						
*No of rRNA genes*	478	256	145	108	545	2126
*No of tRNA genes*	725	801	1115	1007	972	896
*No of miRNA genes*	93	146	231	176	87	89
*No of snoRNA genes*	684	509	1942	687	3798	9350
*No of pseudogenes*	565	868	2760	1553	3202	4688

**Transposable elements**						
*Total size of transposable elements (TEs in Mb)*	270.56	277.95	488.91	194.66	660.71	944.46
*TEs share in the genome*	51.03%	46.73%	48.15%	41.14%	61.89%	69.98%

By integrating homology searches, *ab initio* prediction, and mRNA expression evidence, we predicted a total of 25,105 protein-encoding gene models in chickpea; 29,482 in pigeonpea; 51,839 in soybean; 37,474 in subterranean clover; 33,810 in *A. duranensis*; and 38,783 in *A. ipaensis* (Table 1; Fig. 1**b and 2b**). The average mRNA, CDS, intron, and exon lengths were similar in all of the studied legumes (**Table S4**; Fig. S6). Among studied legumes, the gene count was highest in soybean, a pattern observed in other polyploid crops as well [60], [61]. The majority of predicted genes (>95%) in each of the “C assemblies” were assigned functional annotations using various public databases (**Table S5**). In addition to protein-coding genes, we also identified a range of 87 to 231 miRNA, 108 to 2,126 rRNA, 725 to 1,115 tRNA, and 509 to 9,350 snoRNA genes (**Table S6**). We also investigated pseudogenes in “C assemblies” and found that the maximum number was predicted in *A. ipaensis* (4,688) and the minimum in chickpea (565) (Table 1). The number of pseudogenes in each legume was directly correlated with its genome size.

The “C assemblies” and predicted gene models were evaluated for their completeness using Benchmarking Universal Single-Copy Orthologs [62]. More than 90% of the 1,440 core embryophyta genes were identified in “C assemblies” of all species, indicating the high quality of genome assemblies and annotations (**Table S7**).

### Quality evaluation and improvement of genome assemblies (“D assemblies” vs. “C assemblies”)

We compared the “C assemblies” with the previous “D assemblies” for a range of features to assess quality improvement. In all cases, the “C assemblies” were superior. For instance, the genome sequences anchored to chromosomes in “C assemblies” increased from 40.86% and 65.24% to 91.35% and 92.59% in pigeonpea and chickpea, respectively (**Table S8**). The comparison of Hi-C contact matrices for “C” and “D” assemblies for a given species are indicative of significantly improved “C” assemblies (Fig. 1**a and 2a**). While comparing the improvement in “C assemblies” over “D assemblies”, we found distinct superiority in the new assemblies for the characteristics described below.

*Reconstituting the pseudomolecules:* While comparing the “C assemblies” with the corresponding “D assemblies”, we observed a one-to-one association between pseudomolecules for all legumes except pigeonpea (Figs. S7-S12). In the case of pigeonpea, colinearity was not observed for three pseudomolecules. Therefore, a genetic map containing 6,868 high-quality SNPs and spanning 995.63 cM was constructed based on 336 F_2_ individuals (ICPA 2039 × ICPL 87119) without any guidance from “C” or “D” assemblies (Fig. S13; **Data S1**). A comparison of this map with both assemblies showed an assignment of 97.64% of loci to the 11 appropriate pseudomolecules in the “C assembly” compared to only 64.30% in the “D assembly”. Three pseudomolecules in the “C assembly” that did not have colinearity to the previous assembly were named based on the linkage groups of the genetic map developed. A comparison of the genetic map and Hi-C guided pseudomolecules showed excellent colinearity, indicating better quality of the “C assembly” (Fig. S14; **Data S1**). We found that CcLG02 of the “D assembly” was split into two different pseudomolecules (CcLG02_v2.0 and CcLG05_v2.0), suggesting CcLG02 was incorrectly joined in the “D assembly”. Additionally, CcLG05 and CcLG09 of the “D assembly” are now part of a single pseudomolecule (CcLG09_v2.0) in the “C assembly” (Fig. 2**c and 2d**; Fig. S8). In case of remaining legumes, the pseudomolecules were ordered and oriented based on the respective “D assemblies”.

*Improvement in pseudomolecules:* In “C assemblies” of some legumes such as chickpea, pigeonpea, soybean, and subterranean clover, the length of pseudomolecules was also improved. For instance, in chickpea, the average increment in length across eight pseudomolecules was 45.94%, with a minimum of 12.42% in Ca1_v2.0 to a maximum of 91.86% in Ca3_v2.0. Among all the “C assemblies” developed in the present study, the most significant increment in pseudomolecules length (average 199.10% across 11 pseudomolecules) was observed in pigeonpea. Interestingly, CcLG05_v2.0 has increased by 707.39% compared to the “D assembly”. In contrast, subterranean clover and soybean exhibited average increase of 12.16% across eight pseudomolecules and 1.83% across 20 pseudomolecules length, respectively (**Table S8**).

*Correcting misjoins:* The comparison of “C assemblies” with “D assemblies” highlighted numerous assembly errors (including chimeric joins and small scale to significant chromosome arm-sized inversions) in short-read based “D assemblies” (Figs. S7–S12). The maximum number of errors were identified in the “D assembly” of pigeonpea (4,573), followed by subterranean clover (2,328) and *A. duranensis* (2,192) (**Table S9**). Some of the significant corrections in terms of size included inversions of ∼ 42 Mb and ∼ 19 Mb in pseudomolecules 6 and 7, respectively, of chickpea (Fig. S7)*.* Similarly, inversions of ∼ 19 Mb (pseudomolecule 4) and ∼ 13 Mb (pseudomolecule 9) in the case of *A. duranensis* and ∼ 16 Mb (in pseudomolecules 4 and 10) in *A. ipaensis* were corrected (Figs. S11 and S12).

### Genome organization and evolution of legumes

#### Gene colinearity within a genome and among genomes

Genomic colinearity is a direct reflection of the structure of the ancestral genome. Based on six sets of high-quality genomic data, we were able to identify genomic colinearity (as colinear genes) within each legume genome, between each pair of genomes, and between them and the grape genome, which was used as an outgroup reference. Homologous blocks with more than 4, 10, 20, and 50 colinear genes were identified and recorded (**Tables S10 and S11**).

Among the studied legumes, the number of within-species colinear genes was lowest in subterranean clover. We identified 4,268 colinear genes in 475 duplicated blocks in subterranean clover, each having at least four colinear genes. The subterranean clover genome shared appreciable gene colinearity with the other genomes, having 16,097 (grape) to 45,404 (soybean) colinear genes. As expected, subterranean clover shares the most colinear genes with soybean, which was affected by an extra whole-genome duplication and theoretically doubled the number of colinear blocks with the other legumes (**Table S10**). By characterizing sequence divergence between colinear genes and relating them to different events, polyploidization, or speciation, we managed to infer orthologous and outparalogous genes between different genomes (**Tables S12 and S13**). Outparalogs, produced by polyploidization shared by legumes, would often have higher divergence than orthologs. Among the six studied legumes, subterranean clover has a maximum number (14,414) of colinear orthologs in chickpea, and the two species share 4,034 outparalogs which were duplicated due to the eudicot-common hexaploid (ECH) and legume-common tetraploid (LCT) events that occurred in the common ancestors of both genomes.

Genome-wide comparison of “C assemblies” showed much improved gene colinearity in the six legume genomes. For instance, the number of identified pigeonpea colinear genes showed a >200% increase, from a previously measured 2,086 using the “D assembly” [42] to 6,893 colinear genes found using the “C assembly”, and its colinearity with other genomes also was more than doubled (**Table S10**). Similarly, chickpea colinear genes exhibited nearly 40% increase, from 4,376 to 5,992 and the two wild groundnut diploid genomes showed more than 25% increase. Despite the reduction in total genes predicted in the “C assemblies” (**Table S8**), a significant increase in the number of colinear genes indicated that the increase was a result of improved contiguity of the “C assemblies”.

#### Genome fractionation

After a polyploidization event, many duplicated genes are subsequently lost by the non-random process called genome fractionation [63]. Here, by referring to the grape genome [44] and using the completeness of the present assembled genomes, we found that, in subgenomes produced by the ECH, LCT, or soybean-specific tetraploid (SST) events, often >80% of ancestral genes were deleted from their original location, and about two-thirds of ancestral genes were deleted from two or four copies of homoeologous regions in each genome (**Table S13**). This shows an accumulated effect of genome fractionation after the split from other non-legume eudicots. Moreover, by referring to a legume relative, *Medicago truncatula* [64], we found that about 70% of ancestral genes were deleted in a subgenome, showing genome fractionation after the LCT event that occurred less than 60 million years ago (mya) (**Table S14**).

Gene deletion may occur in a segmental manner. That is, neighboring genes could be deleted from an ancestral chromosomal region at the same time, or accumulated small (e.g., single gene) deletions might result in a similar observation [65]. Here, we used a previously employed statistical approach to find a possible molecular mechanism for these deletions [42]. Compared to a reference genome, grape or *Medicago*, we counted the number of unmatched or non-colinear gene numbers between colinear genes and found that the numbers followed a geometric distribution (**Table S15**), with a further gene deletion rate of ∼0.30–0.42. The further gene deletion rate means that when a DNA strand breaks, DNA deletion has a probability of 0.30–0.42 to extend to involve the following gene. To be more precise, a single gene may be deleted at a probability of 30–42%, two neighboring genes at 9–16%, and three genes at 2.7–6.4%. This suggests that most genes were deleted in relatively short DNA segments.

#### Event-related genomic homology

We attempted to infer homologous genes related to different polyploidization events of speciation by referring to intra- and inter-genomic homologous gene dot plots (Figs. S15–S22). We inferred colinear genes and characterized their molecular divergence levels with Ks (synonymous nucleotide substitution rates), and for homologous blocks within a genome or between genomes, we calculated the median Ks, a relatively stable statistic as compared to mean Ks. The median Ks values can distinguish homologous blocks produced by different events. For example, the older ECH event produced more diverged homologous genes than the LCT or the SST. A few cases were unclear on the origin of homologous blocks due to short blocks with few colinear genes and/or less statistical power in Ks measurement, and complement chromosome segments could be explored to find the truth. Eventually, we inferred event-related colinear genes among genomes with grape or *Medicago* genomes as a reference. In subterranean clover, we inferred 2,216 ECH-produced and 3,612 LCT-produced colinear genes, respectively. The number of identified LCT-produced gene pairs in pigeonpea increased from 464 in the “D assembly” to 2,783 in the “C assembly”. The “C assemblies” of other legumes also showed an increase of these event-related colinear genes as compared to “D assemblies” [42] (**Table S16**).

**Fig. 3:**
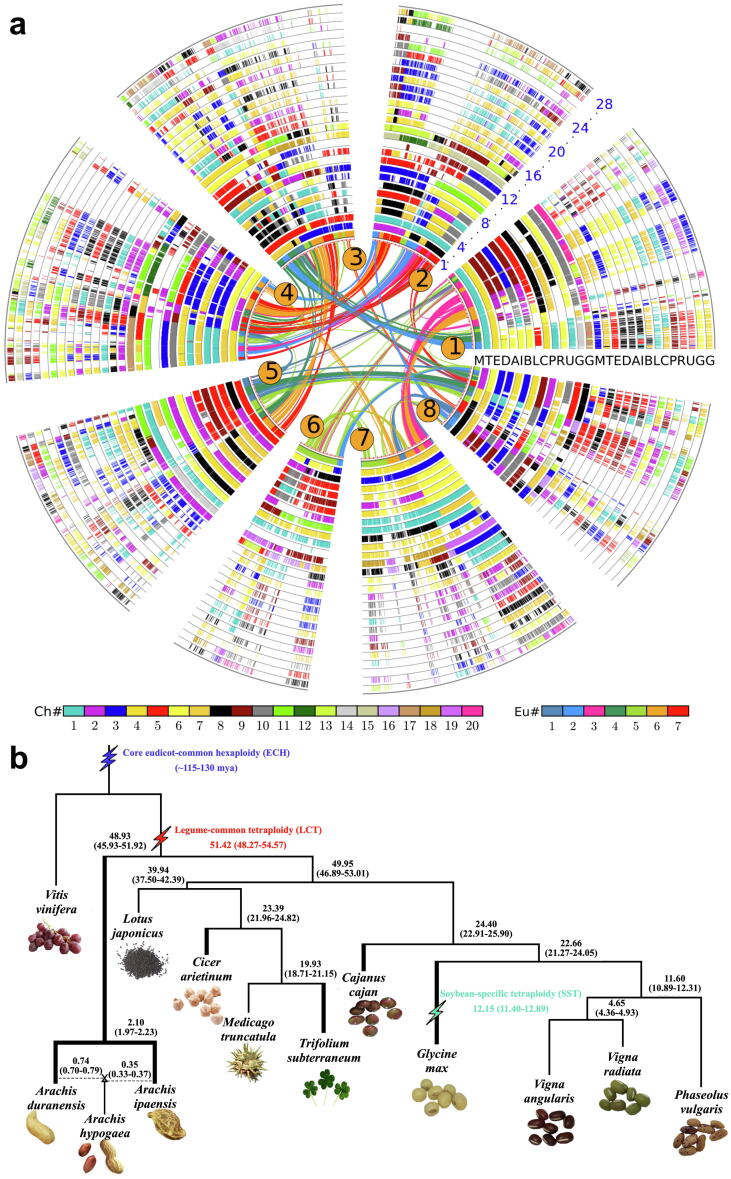
**Evolutionary analysis of different legumes. (a)** Homologous alignments of 13 legume genomes with *Medicago truncatula* as reference. Genomic paralogy, orthology, and outparalogy information within and among 12 legumes were displayed in 28 circles: The curved lines within the inner circle, colored by seven eudicot ancestral chromosomes, denote the linked paralog pairs on eight chromosomes of *M. truncatula* produced by legume-common tetraploidy (LCT). The short lines forming the innermost circles represent all predicted genes in *M. truncatula*, which have one paralogous region, forming another circle. Each of the two sets of *M. truncatula* paralogous chromosomal regions has one orthologous copy in a legume except soybean, which would have two. Cultivated groundnut subgenomes (A and B) were considered as two different species. Therefore, 13 genomes resulted in 28 ((12 + 1x 2) × 2) circles in the figure. Homologous genes are denoted by short lines standing on a chromosome circle and colored as to its chromosome number in the source plant shown in the inset legend. Abbreviations: M, *Medicago truncatula*; T, *Trifolium subterraneum*; E, *Cicer arietinum*; D, *Arachis duranensis*; A, *Arachis hypogaea* A-subgenome; I, *Arachis ipaensis*; B, *Arachis hypogaea* B-subgenome; L, *Lotus japonicus*; C, *Cajanus cajan*; P, *Phaseolus vulgaris*; R, *Vigna radiata*; U, *Vigna angularis*; G, *Glycine max*. **(b)** A phylogenetic tree of 12 legume species with grape as an outgroup species. Thick branches show legume species with improved genome assemblies in this study. A blue flash marks the major-eudicot-common hexaploidy (ECH), a red one the LCT, and a yellow one the soybean-specific tetraploidy (SST). The divergence time (in million years ago) of each species is mentioned in the tree. (For interpretation of the references to color in this figure legend, the reader is referred to the web version of this article.)

With these event-related genes among genomes, we performed multiple genome alignment, which provided a direct display of genome fractionation in each genome, and to each event. For example, with the grape genome as the reference, we produced two genome alignments. The first one shows all the orthologous and outparalogous genes that were mapped onto the grape genome (Fig. S23). That is, for a grape gene, all its legume colinear orthologs were revealed, and all the ECH colinear outparalogs were inferred. The second one shows only the orthologous genes (Fig. S24), showing the correspondence between the extant legume and grape chromosomes. The alignment with the *Medicago* genome as reference was also constructed similarly to show relatedness between legume chromosomes (Fig. 3**a**; Fig. S25). The alignments provide valuable information to distinguish orthologs and outparalogs, involving thousands of simultaneously originated homologs in an evolutionary event (polyploidization or speciation), especially with much improved assemblies, laying a solid foundation to explore the origin, evolution, and functional innovation in genes and regulatory pathways.

#### Evolutionary dating

With colinear genes identified in the “C assemblies”, we dated the speciation between legumes and the polyploid events that occurred by using a normal fitting distribution. Using the ECH event at 115–130 mya [66], as a standard, we dated numerous legume events (Fig. S26; **Table S17**). The occurrence of the LCT at ∼ 51.42 mya (48.27–54.57) agrees with previous estimates by different groups [42]. Our results indicate that the groundnut (dalbergioid) lineages split from the other legumes ∼ 48.93 mya (45.93–51.92). Only about one million years later, the hologalegina (chickpea, lotus (*Lotus japonicus*), *Medicago*, subterranean clover) and indigoferoid/millettioid (adzuki bean (*Vigna angularis*), common bean (*Phaseolus vulgaris*), mungbean (*Vigna radiata*), pigeonpea, soybean) lineages split to form two large legume subgroups. The subterranean clover lineage split from the *Medicago* lineage ∼ 19.93 mya (18.71–21.15). As per our analysis, the SST occurred less than 12.15 mya (11.40–12.89) because the dating of ancestral genome divergences provides a maximum time after which the polyploidy occurred. In the case of *Arachis* species, while Ad (*A. duranensis*) and Bd (*A. ipaensis*) genome lineages were calculated to have diverged 2.10 (1.97–2.23) mya, the divergence of Ad-At (At, A-subgenome of cultivated groundnut) and Bd-Bt (Bt, B-subgenome of cultivated groundnut) appears to have occurred about 0.74 (0.70–0.79) and 0.35 (0.33–0.37) mya, respectively (Fig. 3**b**)**.** These latter values support the proposal by Zhuang and colleagues [38], [67] but not the more recent origin suggested by Bertioli and colleagues [68], [69].

### Understanding legume biology based on gene family analysis

**Fig. 4:**
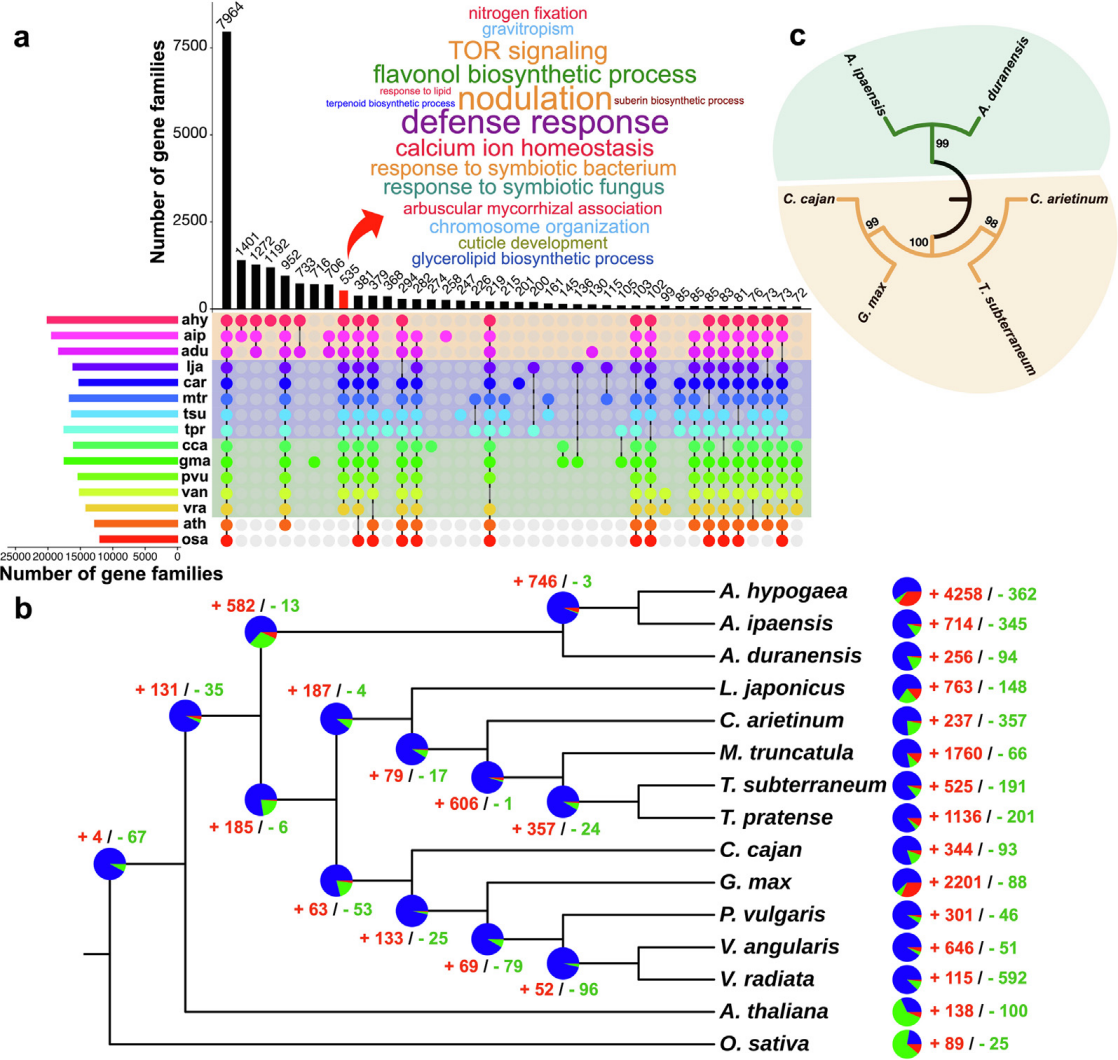
**Gene conservation and gene family expansion and contraction analysis. (a)** An UpSet plot depicting the number of orthogroups shared between different species. The red bar highlights the legume-specific gene families and the significantly (*P*-value < 0.05) enriched GO terms in these families. The top 40 overlaps based on frequency are plotted. The horizontal bars represent the number of gene families per species. The rows are highlighted based on clade (red, dalbergoid; blue, galegoid; green, milletioid). Species abbreviations: adu, *Arachis duranensis*; ahy, *Arachis hypogaea*; aip, *Arachis ipaensis*; ath, *Arabidopsis thaliana*; car, *Cicer arietinum*; cca, *Cajanus cajan*; gma, *Glycine max*; lja, *Lotus japonicus*; mtr, *Medicago truncatula*; osa, *Oryza sativa*; pvu, *Phaseolus vulgaris*; tpr, *Trifolium pratense*; tsu, *Trifolium subterraneum*; van, *Vigna angularis*; vra, *Vigna radiata*. **(b)** Estimation of gene family expansion and contraction in different legumes. The species tree was constructed based on single-copy orthologs. The pie-charts represent the number of expanded (in red), contracted (in green), and unchanged (in blue) gene families. The numbers next to the pie charts denote the number of significantly (*P*-value < 0.05) expanded (in red) and contracted (in green) gene families. **(c)** A phylogenetic tree for the legumes (*A. duranensis*, *A. ipaensis*, *C. arietinum*, *C. cajan*, *G. max*, and *T. subterraneum*) based on the concatenated sequences of nodulation genes. The two different clades are highlighted in orange and green. Protein sequences were aligned using ClustalW in MEGA X software. Bootstrap values are indicated in the tree (based on 1,000 bootstrap replications). (For interpretation of the references to color in this figure legend, the reader is referred to the web version of this article.)

Predicted proteins from the six newly annotated genomes were compared to those already annotated in other members of the Papilionoideae family, including *Medicago* [63], lotus [70], adzuki bean [71], common bean [72], mungbean [73], cultivated groundnut [68] and red clover (*Trifolium pratense*) [74], to identify unique and shared gene families between different species using *Arabidopsis* and rice (*Oryza sativa*) as the outgroup species. Reciprocal pairwise comparisons of the proteins of 15 species led to the identification of 36,854 gene families (**Table S18**). The analysis identified 535 gene families that are specific to Papilionoideae (Fig. 4**a**). These families were enriched in genes involved in defense response, nodulation, TOR signaling, flavonol biosynthesis, calcium ion homeostasis, response to symbiotic bacterium, arbuscular mycorrhizal association, nitrogen fixation, and gravitropism (Fig. 4**a**). Within the studied legumes, the number of gene families specific to galegoid (chickpea, lotus, *Medicago*, red clover, and subterranean clover), milletioid (adzuki bean, common bean, mungbean, pigeonpea, and soybean), and dalbergioid (cultivated groundnut, *A. duranensis*, and *A. ipaensis*) lineages was 85, 72, and 1,272, respectively (Fig. 4**a**). Cultivated groundnut shared a higher number of gene families with its B-progenitor (*A. ipaensis*; 1,401) as compared to its A-progenitor (*A. duranensis*; 733).

The identified gene families were studied for expansion/contraction during legume evolution. A total of 131/35 families were significantly expanded/contracted in Papilionoideae (*P*-value < 0.05; Fig. 4**b**). In the case of chickpea, 237 and 357 families were considerably expanded and contracted, respectively. Functional annotation of expanded gene families suggested enrichment for genes involved in short-day photoperiodism, cellulose catabolism, xyloglucan biosynthesis, and red, far-red light phototransduction. The members of dalbergioid clade and soybean demonstrated expansion of genes mainly involved in oil biosynthesis (fatty acid, lipid, diterpenoid, and flavonoid biosynthetic processes), auxin biosynthesis, and gravitropism (**Data S2**).

Nodulation is a characteristic feature of legumes that can furnish them with a competitive growing advantage in nitrogen-poor soils compared with non‐legume plants. Multiple genes are involved in the formation and development of root nodules. We identified and investigated such genes in the studied legumes (99 in chickpea, 105 in pigeonpea, 129 in soybean, 98 in subterranean clover, 93 in *A. duranensis*, and 93 in *A. ipaensis*; **Table S19**). The species tree based on concatenated sequences of nodulation-related genes highlighted that both the *Arachis* species formed one clade, and remaining legumes were part of another clade indicating the presence of a unique nodulation mechanism among the members of *Arachis* species and needs to be investigated further (Fig. 4**c**).

Resistance to a wide array of pathogens and pests, such as bacteria, fungi, viruses, insects, and nematodes, is a pivotal contributor to crop yield. Part of disease resistance in plants is contributed by different plant resistance gene analogs (RGAs) such as NBS-encoding proteins, receptor-like proteins (RLPs), and receptor-like protein kinases (RLKs). Among the studied legumes, the number of RGAs ranged from 828 in chickpea to 1,698 in soybean (**Table S20**). Among the identified RGAs, RLKs were the most abundant class, followed by NBS-encoding genes. We identified 140 (in chickpea) to 532 (in subterranean clover) NBS-encoding genes in studied genomes. In chickpea (35.00%), pigeonpea (29.74%), subterranean clover (24.06%), *A. duranensis* (34.91%), and *A. ipaensis* (21.59%), a high number of RGAs were CC-NBS-LRR (CNL) genes in contrast to soybean (21.91%), where TIR-NBS-LRR (TNL) genes were most abundant (**Table S21**). In chickpea, of the total NBS-encoding genes, 137 (97.86%) were mapped to one of the eight pseudomolecules with significantly biased distribution among the pseudomolecules (Chi-squared test *P*-value < 1E-08); ∼33.57% were located on pseudomolecule Ca5_v2.0 (**Table S22**; Fig. S27). Similar patterns of biased distribution were observed in all legumes where CcLG09_v2.0 of pigeonpea (20.51%), Tr_Chr3_v2.0 of subterranean clover (21.62%), Gm.Lee_Chr06_v2.0 (10.76%) of soybean, Aradu_Chr02_v2.0 (39.62%) of *A. duranensis*, Araip_Chr02_v2.0 and Araip_Chr04_v2.0 of *A. ipaensis* (20.93% and 18.60%, respectively) harbored a significantly high number of NBS genes (Figs. S28-S32).

An average of 2,282 putative transcription factors (TFs) belonging to 58 families were predicted from the six legumes. These TFs constitute 5.17% (subterranean clover) to 7.16% (soybean) of the predicted protein-coding genes (**Table S23**). In each of the studied legumes, bHLH, MYB, ERF, FAR1, C2H2, WRKY, and NAC were the most abundant TF families (Fig. S33). Interestingly, an *Arachis*-specific expansion of FAR1 TFs was seen, with 254 TFs in *A. duranensis,* and 445 in *A. ipaensis.* These results support the previous findings that FAR1 TFs might have implications in the process of geocarpy (characteristic of *Arachis* genus), given their role in the regulation of skotomorphogenesis and photomorphogenesis in higher plants [75].

### “C assemblies” provided novel genes for crop improvement

To assess the advantage of “C assemblies” over “D assemblies” in detection of genes/genomic segments associated with agronomically important traits, studies were conducted in chickpea and pigeonpea. Specific cases are described below.

#### High-resolution mapping of drought tolerance in chickpea

**Fig. 5:**
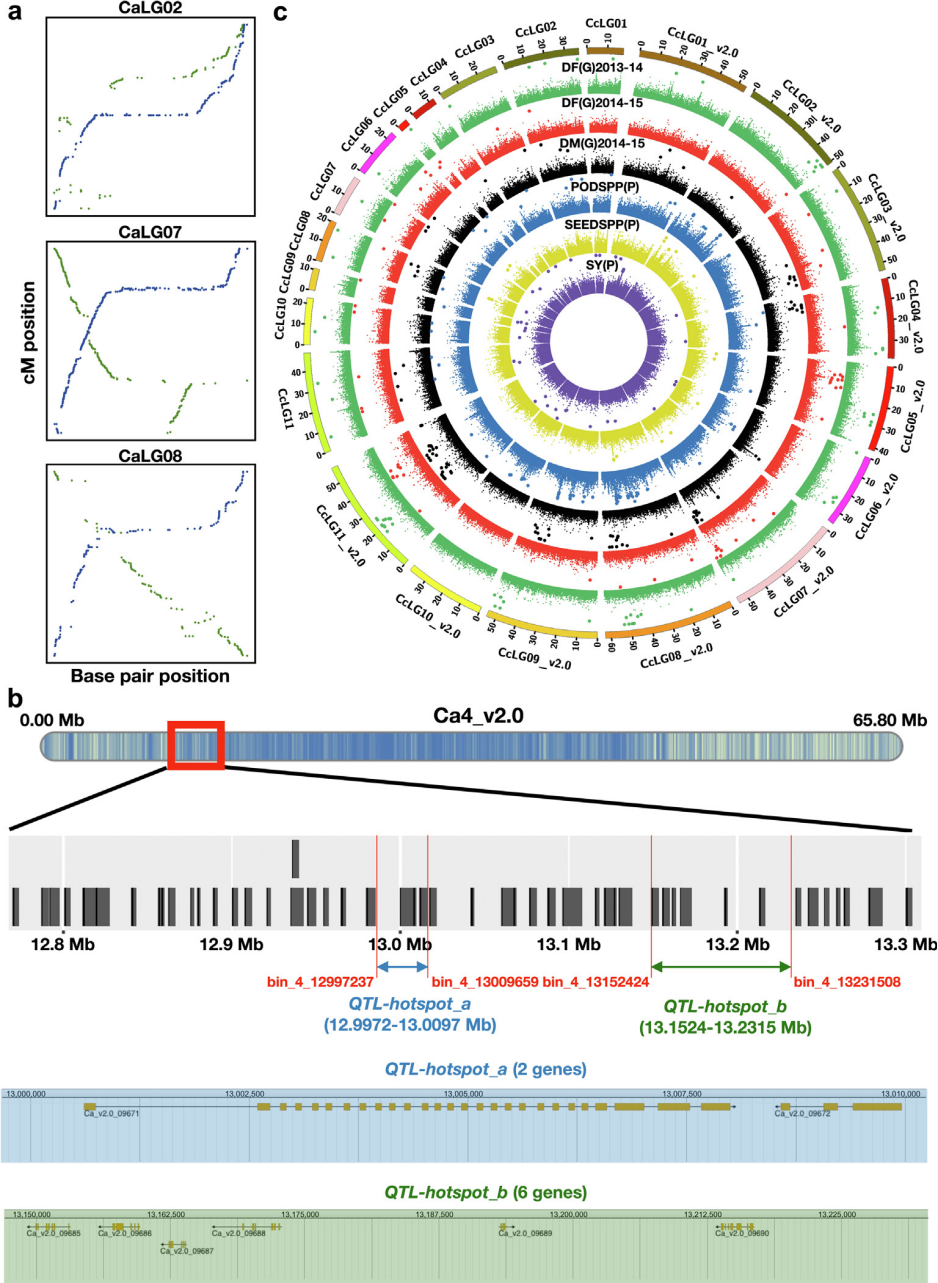
**Examples of utilization of improved genome assemblies for genetics research and breeding applications in chickpea and pigeonpea. (a)** Improvement of correlation between genetic map and genome assembly as shown in three pseudomolecules of chickpea (CaLG02, CaLG07, and CaLG08) in the “D” (green dots) and “C” (blue dots) assemblies. The x-axis and y-axis represent the coordinates of genome assembly and genetic map, respectively. **(b)** High-resolution mapping of the “*QTL-hotspot*” region. The color scale on the ideogram denotes the gene density. The black bars on the zoomed-in region represent the genes. The flanking markers for the re-defined “*QTL-hotspot*” regions are highlighted in red. The “*QTL-hotspot_a*” and “*QTL-hotspot_b*” and the candidate genes in these regions are highlighted in blue and green, respectively. **(c)** A circos diagram illustrating the improved marker-trait associations (MTAs) identified through GWAS analysis performed on both “D” and “C” assemblies of pigeonpea. The significant MTAs (*P*-value < 1E-05) are highlighted with bigger dots. Abbreviations: DF, days to 50% flowering; DM, days to 75% maturity; PODSPP, pods per plant; SEEDSPP, seeds per plant; SY, seed yield; G, Gulbarga; P, Patancheru. (For interpretation of the references to color in this figure legend, the reader is referred to the web version of this article.)

In order to make chickpea a more resilient crop, a “*QTL-hotspot*” region for drought tolerance has been identified in one recombinant inbred line (RIL) population developed from the ICC 4958 (drought-tolerant) × ICC 1882 (drought-sensitive) cross [47]. For dissecting this “*QTL-hotspot*” region, each RIL was sequenced at 1X coverage, and by aligning these sequencing data with the “D assembly”, a recombination breakpoints-based genetic map was developed with 53,223 SNPs in 1,610 bins [48]. QTL analysis based on this map together with 17 drought tolerance-related traits identified 71 significant QTL, including splitting of the “*QTL-hotspot*” region into two sub-regions namely “*QTL-hotspot*_*a*” (139.22 kb) and “*QTL-hotspot*_*b*” (153.36 kb). To assess the utility of the “C assembly” for enhancing the resolution of QTLs, the sequencing data of the population was mapped to the “C assembly” that provided 85,598 high-quality SNPs (∼61% higher SNPs identified as compared to the “D assembly”). Based on these data, an improved recombination breakpoints-based genetic map with 2,495 bins and spanning 700.14 cM genetic distance was developed (Fig. S34; **Data S3**). This improved bin map showed higher colinearity with the genome assembly compared to the earlier bin map developed using the “D assembly” (Fig. 5**a**; Fig. S35**)**. Moreover, the new bin map shows expected properties, like a low recombination rate in centromeric regions while higher recombination in telomeric regions. QTL analysis by using this bin map and the above-mentioned phenotyping data identified 137 QTL, including 40 major QTL as compared to 71 major QTL described previously [48]. Furthermore, the major effect QTL on CaLG08, reported by Kale et al. [48] were identified as minor QTL in this study, in accordance with Varshney et al. [47]. Therefore, bin map developed based on the “C assembly” was useful in removing spurious QTL detected with the bin map developed using the “D assembly”.

In the present study, the topmost QTL for each trait were located in two hotspot regions, 12997237–13009659 (12.42 kb) and 13152424–13231508 (79.08 kb) on Ca4_v2.0 in the “C assembly” (Fig. 5**b**). These regions are exactly colinear with 13158245–13170667 (12.42 kb) and 13313432–13392516 (79.08 kb) regions on Ca4 in the “D assembly”. While analysing “*QTL-hotspot_a*” (139.22 kb) and “*QTL-hotspot_b*” (153.36 kb) with the “C assembly”, we identified 68.87 kb upstream region of “*QTL-hotspot_a*” matching with 12.42 kb (12997237–13009659), and 1.13 kb upstream region of “*QTL-hotspot_b*” matching with 79.08 kb (13152424–13231508) in the “C assembly”, which coincides with the “*QTL-hotspot_a*”. Therefore, the QTL analysis using bin map based on “C assembly” shortened the sizes of “*QTL-hotspot_a*” from 139.22 kb to 12.42 kb and “*QTL-hotspot_b*” from 153.36 kb to 79.08 kb (48.43%). The re-defined “*QTL-hotspot*” region harbored eight candidate genes compared to 26 genes in the previous “*QTL-hotspot*” region (Fig. 5**b**). This detailed analysis indicated the presence of two additional genes (*Ca_v2.0_09671* and *Ca_v2.0_09672*) in the re-defined “*QTL-hotspot*” region. Functional annotation suggested the eight candidate genes mainly encoded for stress-responsive proteins such as serine/threonine-protein kinase, TIFY 4A-like, and epidermal patterning factor.

#### Novel genomic segments for yield-related traits in pigeonpea

Yield is one of the most important, variable and complex traits across crop species. It can be enhanced many fold by improving yield attributing or contributing traits. In order to discover genes/genomic segments associated with yield attributing traits in pigeonpea, a genome-wide association study (GWAS) was performed with whole-genome sequencing (WGS) data and multi-location/years traits phenotyping data on 292 pigeonpea lines. To compare the utility of the “C assembly” over the “D assembly” for GWAS analysis, WGS data on 292 pigeonpea lines [52] were used for variant detection using both the “C” and “D” assemblies of pigeonpea as references. We identified over 7.97 million high-quality SNPs from the “C assembly”, numbers significantly higher than the high-quality SNPs (6.68 million) identified from the “D assembly” (**Table S24**). After identifying genome-wide SNPs across pigeonpea lines, SNP loci with minor allele frequencies less than 5% and more than 20% heterozygosity were eliminated from the analysis. Therefore, 731,585 SNPs and 317,120 SNPs from the “C” and “D” assemblies, respectively, were used for GWAS with phenotyping data for nine agronomic traits collected from three locations over two years. In total, 132 marker-trait associations (MTAs) with the “C assembly” and 97 MTAs with the “D assembly” were identified (Fig. 5**c**; **Table S25**). Out of 132 MTAs identified with the “C assembly”, 82 were located on newly assembled contigs (i.e., they were free-floating contigs in C.cajan_V1.0). Of the total MTAs detected in the “C” and “D” assemblies, three and two MTAs were found to be associated with more than one trait, respectively (**Table S26**). From these three MTAs identified from “C assembly”, two MTAs, i.e., CcLG01_v2.0pos43089516.1 and CcLG08_v2.0pos9028689.1 were found to be associated with days to 50% flowering (DF) and days to 75% maturity (DM), and one MTA CcLG10_v2.0pos11613531.1 was associated with the number of pods per plant (PODSPP) and seed yield (SY). Interestingly, all three of these MTAs in the “C assembly” were located on newly assembled contigs. The only MTA (CcLG05_v2.0pos35533065.1) found consistent across two years at one location for DF was identified with the “C assembly”. The remaining MTAs identified through the “C assembly” and all MTAs identified through “D assembly” were associated with only one dataset for target traits. From the above mentioned total MTAs, 10 MTAs were identified with both genome assemblies. Further, the “C assembly” with newly assembled contigs has discovered new functional variants associated with traits. For instance, MTA CcLG01_v2.0pos32391886.1 detected on CcLG01_v2.0, associated with DF, causing missense mutation was present on the unassembled Scaffold129730 in the “D assembly”. Similarly, CcLG11_v2.0pos28601229.1 associated with the number of seeds per pod (SEEDSPP) causing missense mutation was present on the unassembled Scaffold137823 in “D assembly”. These old and new MTAs identified and mapped with the “C assembly” will be valuable in developing high yielding and early maturing varieties in pigeonpea.

## Discussion

A high‐quality reference genome is pre-requisite to understand genome organization, to describe evolutionary events and to precisely identify genomic regions/genes associated with agronomically important traits. Therefore, in the present study, we have improved reference genomes of six legume species, namely, chickpea, pigeonpea, soybean, subterranean clover, *A. duranensis*, and *A. ipaensis* using the Hi-C analysis. Hi-C is a popular approach for studying how genomes fold inside the nucleus in 3D and has been used to improve genome assemblies of several crop species [76], [77], [78], [79]. The quality of our “C assemblies” are considerably better than the previously published draft genomes [3], [4], [5], [6], [7], as reflected by scaffold N50, BUSCO completeness, and percentage of sequences anchored to pseudomolecules. Nevertheless, it is also important to mention that these assemblies might still contain some errors. Hi-C data provides extensive links covering large distances, but it is not ideal for the local ordering of small adjacent contigs and may require support of additional data [10].

Hi-C relies on the density and proximity of cross-linked chromatin interactions to orient and order the contigs, and it can resolve the errors introduced due to the limitations of genetic maps [80]. For instance, in pigeonpea, Hi-C data corrected the misannotations of pseudomolecules caused by misjoins in the draft genome assembled using SSR-based genetic maps. The “C assemblies” of chickpea and pigeonpea demonstrated a much higher consistency with genetic maps than “D assemblies”. Our study has demonstrated that a high-quality genome assembly is indispensable for the accurate prediction of the gene repertoire. In pigeonpea, a significant reduction of ∼ 40% gene models was seen, suggesting that the gene number was inflated in the draft genome as it might have included genes split across contigs.

Better genome assemblies and better gene colinearity ensure the inference of thousands of credible homologs produced in an evolutionary event, polyploidization or speciation. Homologs in colinearity were much likely produced simultaneously in the corresponding event. This provides a precious opportunity to determine if divergently evolved genes were the ones under natural selection [81]. It was recently reported that many duplicated cotton genes, produced by a *Gossypium*-common decaploidization [82], evolved in much divergent, often elevated, rates [83]. This resulted in aberrant topology that was incongruent with the expected relationship, clearly supported by the gene colinearity. Here, the actual phylogenetic relationship of the inferred legume colinear genes was well indicated by the reconstructed cross-genome alignment, laying a solid foundation to perform evolutionary and functional analysis. Our study suggested that the origin and eventual establishment of legumes, the third largest land plant group, should be related to the LCT, having occurred 51.42 mya. After the event, nearly 18,000 species and 680 genera emerged, making them one of the most successful plant groups. Grasses form another large land plant group, ∼10,000 species in ∼ 620 genera, and their establishment could be related to a tetraploidization ∼ 100 mya [84]. Comparatively, the legumes have expanded about 3.6 times faster than the grasses.

Reference-based variant detection methods are vastly dependent on the quality of the reference genome used for variant calling because the artifacts present in the assembly are passed on to the variants called using them. In this study, we identified more SNPs (19.31%) and MTAs (36.08%) with the “C assembly” as compared to the “D assembly” of pigeonpea that helped improve GWAS results for yield and yield-related traits. Similar observations were reported for blueberry (*Vaccinium corymbosum*), where a more contiguous assembly provided a higher number of significant SNPs with enhanced precision [85]. Furthermore, genetic analysis of the “*QTL-hotspot*” region with the new assembly delimited ∼300 kb region to ∼235 kb and prioritised candidate genes from 26 to 8. It could be attributed to more anchored sequences in the respective region in the “C assembly” thereby increasing the number of markers and recombination bins compared to the “D assembly”.

We have made all datasets reported here public via an online repository - “Legumepedia”. It is freely available at “https://cegresources.icrisat.org/legumepedia/index.php”. Legumepedia is designed to be highly interactive, adaptive, and expandable. We have incorporated the genome assemblies, predicted gene models, and annotations for all of the legumes presented in the current study. A user can use the ‘search’ option to retrieve information about any gene/locus. The repository offers JBrowse, a visualization tool to view the different genomic features of each of the six genomes.

## Conclusion

In summary, this study reports high-quality genome assemblies and genome features of six legume species and demonstrates their utility for basic genetics research and plant breeding applications. The chromosome-length assemblies of these legumes amplify the genomic resources available to the legume community and are potential springboards for accelerating crop improvement via genomics-assisted breeding or genome editing technologies such as CRISPR.

## Ethical statement

This article does not contain any studies with human or animal subjects.

## CRediT authorship contribution statement

**Vanika Garg:** Formal analysis, Investigation, Writing – original draft, Writing – review & editing. **Olga Dudchenko:** Formal analysis, Investigation. **Jinpeng Wang:** Formal analysis, Investigation. **Aamir W. Khan:** Formal analysis, Investigation. **Saurabh Gupta:** Formal analysis, Investigation. **Parwinder Kaur:** Resources, Funding acquisition. **Kai Han:** Formal analysis. **Rachit K. Saxena:** Formal analysis. **Sandip M. Kale:** Formal analysis. **Melanie Pham:** Investigation. **Jigao Yu:** Formal analysis. **Annapurna Chitikineni:** Resources. **Zhikang Zhang:** Formal analysis. **Guangyi Fan:** Formal analysis. **Christopher Lui:** Investigation. **Vinodkumar Valluri:** Formal analysis. **Fanbo Meng:** Formal analysis. **Aditi Bhandari:** Formal analysis. **Xiaochuan Liu:** Formal analysis. **Tao Yang:** Resources. **Hua Chen:** Resources. **Babu Valliyodan:** Resources. **Manish Roorkiwal:** Formal analysis. **Chengcheng Shi:** Formal analysis. **Hong Bin Yang:** Resources. **Neva C. Durand:** Investigation. **Manish K. Pandey:** Formal analysis. **Guowei Li:** Resources. **Rutwik Barmukh:** Formal analysis. **Xingjun Wang:** Resources. **Xiaoping Chen:** Resources. **Hon-Ming Lam:** Resources. **Huifang Jiang:** Resources. **Xuxiao Zong:** Resources. **Xuanqiang Liang:** Resources. **Xin Liu:** Resources. **Boshou Liao:** Resources. **Baozhu Guo:** Resources. **Scott Jackson:** Resources. **Henry T. Nguyen:** Resources. **Weijian Zhuang:** Resources, Funding acquisition. **Wan Shubo:** Resources, Supervision. **Xiyin Wang:** Supervision, Funding acquisition. **Erez Lieberman Aiden:** Supervision, Funding acquisition. **Jeffrey L. Bennetzen:** Writing – review & editing, Supervision. **Rajeev K. Varshney:** Conceptualization, Supervision, Funding acquisition, Writing – original draft, Writing – review & editing.

## Declaration of Competing Interest

The authors declare that they have no known competing financial interests or personal relationships that could have appeared to influence the work reported in this paper.

## Data Availability

The sequencing data and genome assemblies have been deposited in NCBI with the BioProject ID PRJNA679437 and PRJNA512907 (SRR14657175). The genome assemblies and annotations are available at Legumepedia (https://cegresources.icrisat.org/legumepedia/index.php). The interactive Hi-C contact maps for all the six legume genomes are available at www.dnazoo.org.
