# Supplementary material for: Chromosome-length genome assemblies of six legume species provide insights into genome organization, evolution, and agronomic traits for crop improvement
Source: J Adv Res. 2021 Nov 3;42:315–29. doi: 10.1016/j.jare.2021.10.009 (PMC9788938; doi:10.1016/j.jare.2021.10.009)
Supplement: Supplementary data 1 [file mmc1.docx]

**
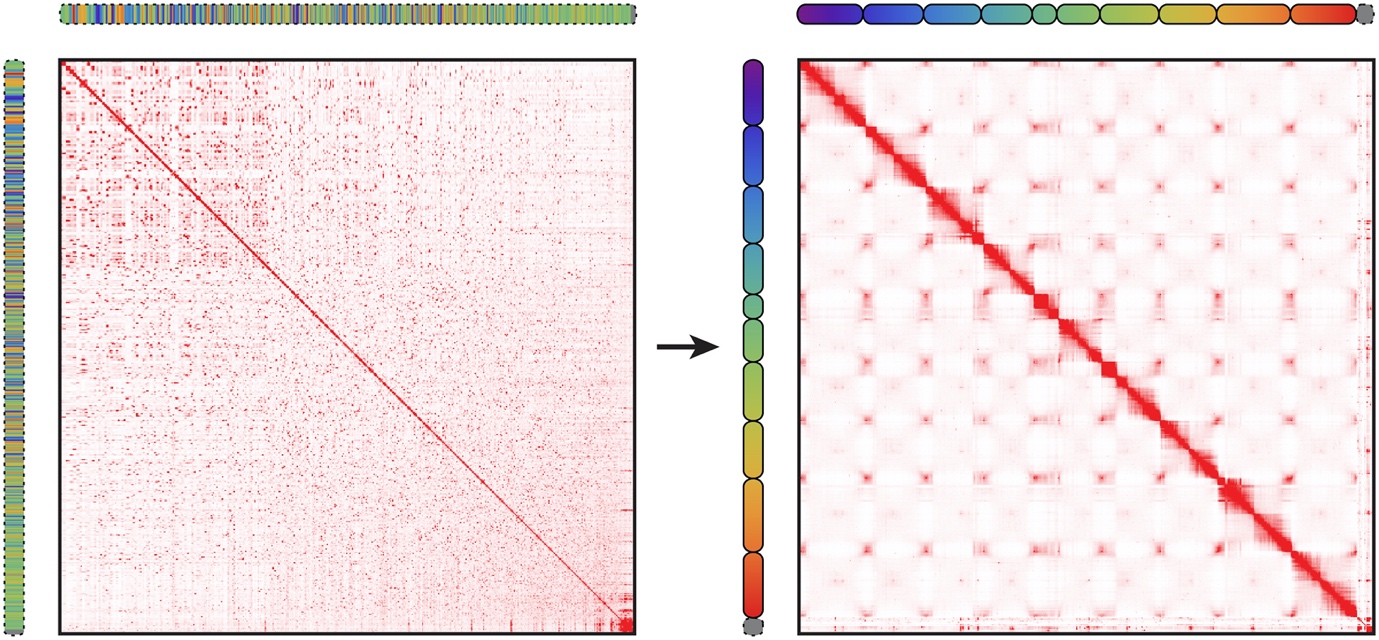
**

**Fig. S1. Hi-C guided assembly of *Arachis duranensis*.** The contact matrices were generated by aligning the same Hi-C data to “D” (Aradu1.1; left) and Hi-C guided “C” (Aradu.V14167_v2.0; right) assemblies. The color intensity in the matrices indicate number of reads supporting co-localisation of a pair of loci in the nucleus. The chromograms show the correspondence between loci in the “D” and “C” assemblies. Scaffolds greater than 10 kb are plotted in this illustration.


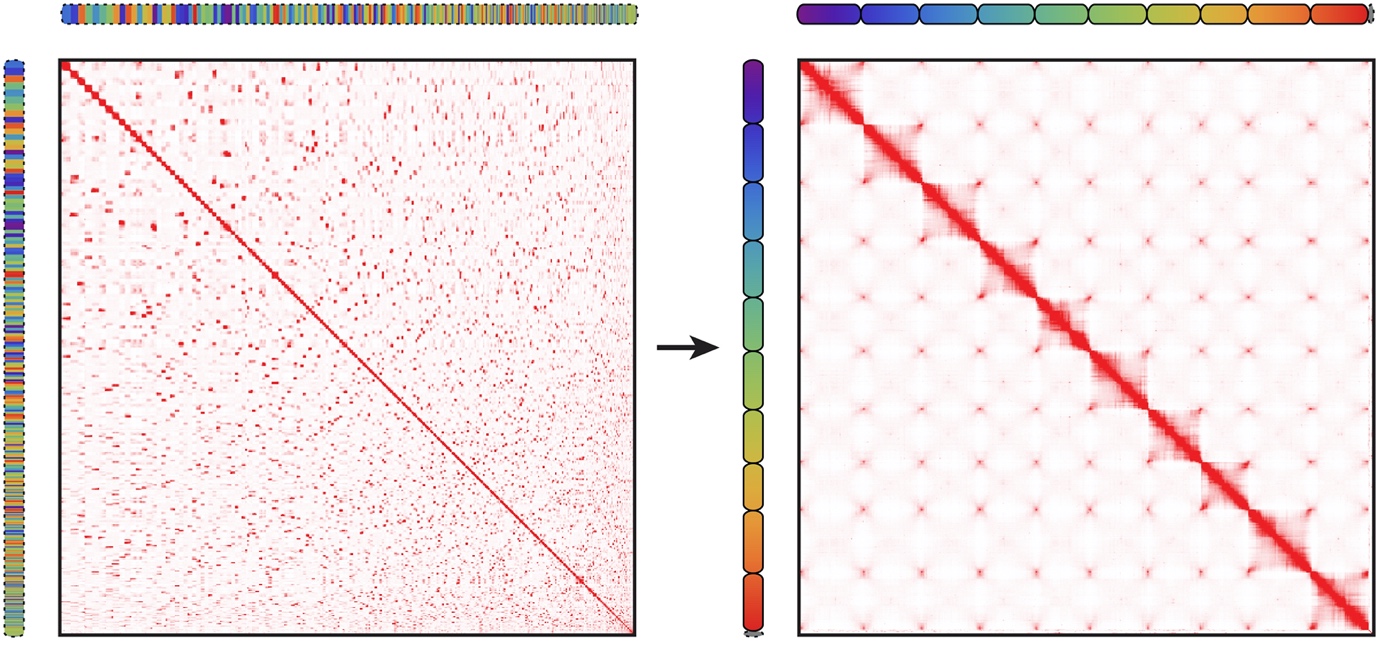


**Fig. S2**. **Hi-C guided assembly of *Arachis ipaensis*.** The contact matrices were generated by aligning the same Hi-C data to “D” (Araip1.1; left) and Hi-C guided “C” (Araip.K30076_v2.0; right) assemblies. The color intensity in the matrices indicate number of reads supporting co-localisation of a pair of loci in the nucleus. The chromograms show the correspondence between loci in the “D” and “C” assemblies. Scaffolds greater than 10 kb are plotted in this illustration.


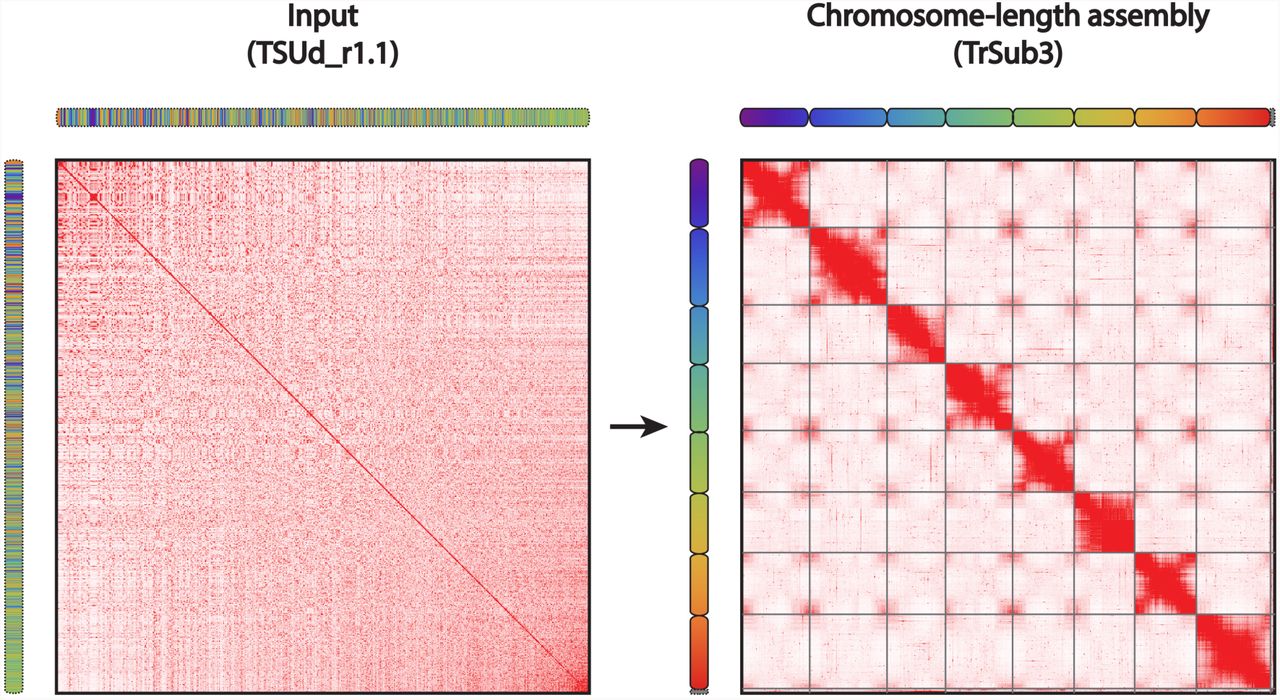


**Fig. S3**. **Hi-C guided assembly of subterranean clover (*Trifolium subterraneum*).** The contact matrices were generated by aligning the same Hi-C data to “D” (TSUd_r1.1; left) and Hi-C guided “C” (Trisu.Daliak_v2.0; right) assemblies. The color intensity in the matrices indicate number of reads supporting co-localisation of a pair of loci in the nucleus. The chromograms show the correspondence between loci in the “D” and “C” assemblies. Scaffolds greater than 10 kb are plotted in this illustration.


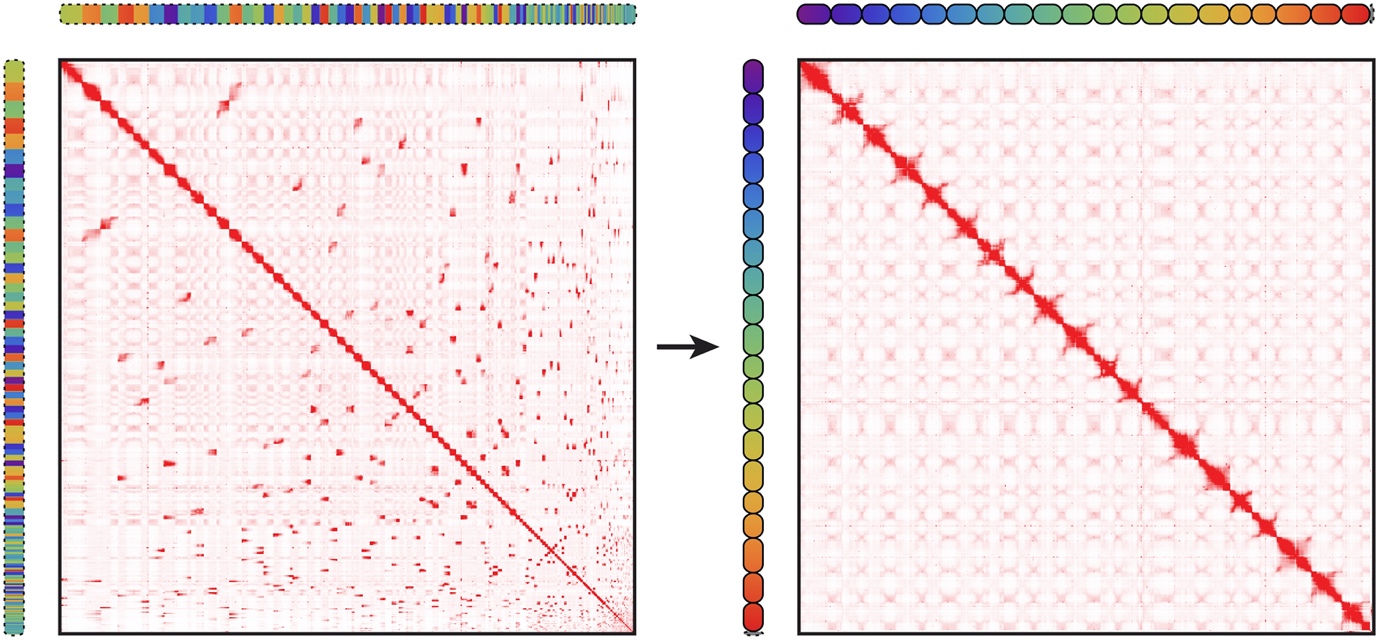


**Fig. S4**. **Hi-C guided assembly of soybean (*Glycine max*).** The contact matrices were generated by aligning the same Hi-C data to “D” (glyma.Lee.gnm1; left) and Hi-C guided “C” (Glyma.Lee_v2.0; right) assemblies. The color intensity in the matrices indicate number of reads supporting co-localisation of a pair of loci in the nucleus. The chromograms show the correspondence between loci in the “D” and “C” assemblies. Scaffolds greater than 10 kb are plotted in this illustration.

**Fig. S5. GC content distribution in the studied legumes.** The mean GC content is plotted in 500 bp windows. The mean GC content of wild groundnut relatives (*Arachis duranensis* and *Arachis ipaensis*) is higher than those of chickpea (*Cicer arietinum*), pigeonpea (*Cajanus cajan*), soybean (*Glycine max*), and subterranean clover (*Trifolium subterraneum*).

**Fig. S6.** **Length distribution of CDS, exons, introns, and mRNA in the studied legumes.** The x-axis and the y-axis represent the length and percentage of (a) CDS, (b) Exons, (c) Introns, and (d) mRNA in the corresponding length window, respectively.

**Fig. S7. Dot plot showing the whole genome alignment between the draft “D” assembly and the Hi-C guided “C” assembly of chickpea (*Cicer arietinum*).** The color of the dots represent the percentage identity where green and yellow denote high and low percentage identity, respectively.

**Fig. S8. Dot plot showing the whole genome alignment between the draft “D” assembly and the Hi-C guided “C” assembly of pigeonpea (*Cajanus cajan*).** The color of the dots represent the percentage identity where green and yellow denote high and low percentage identity, respectively.


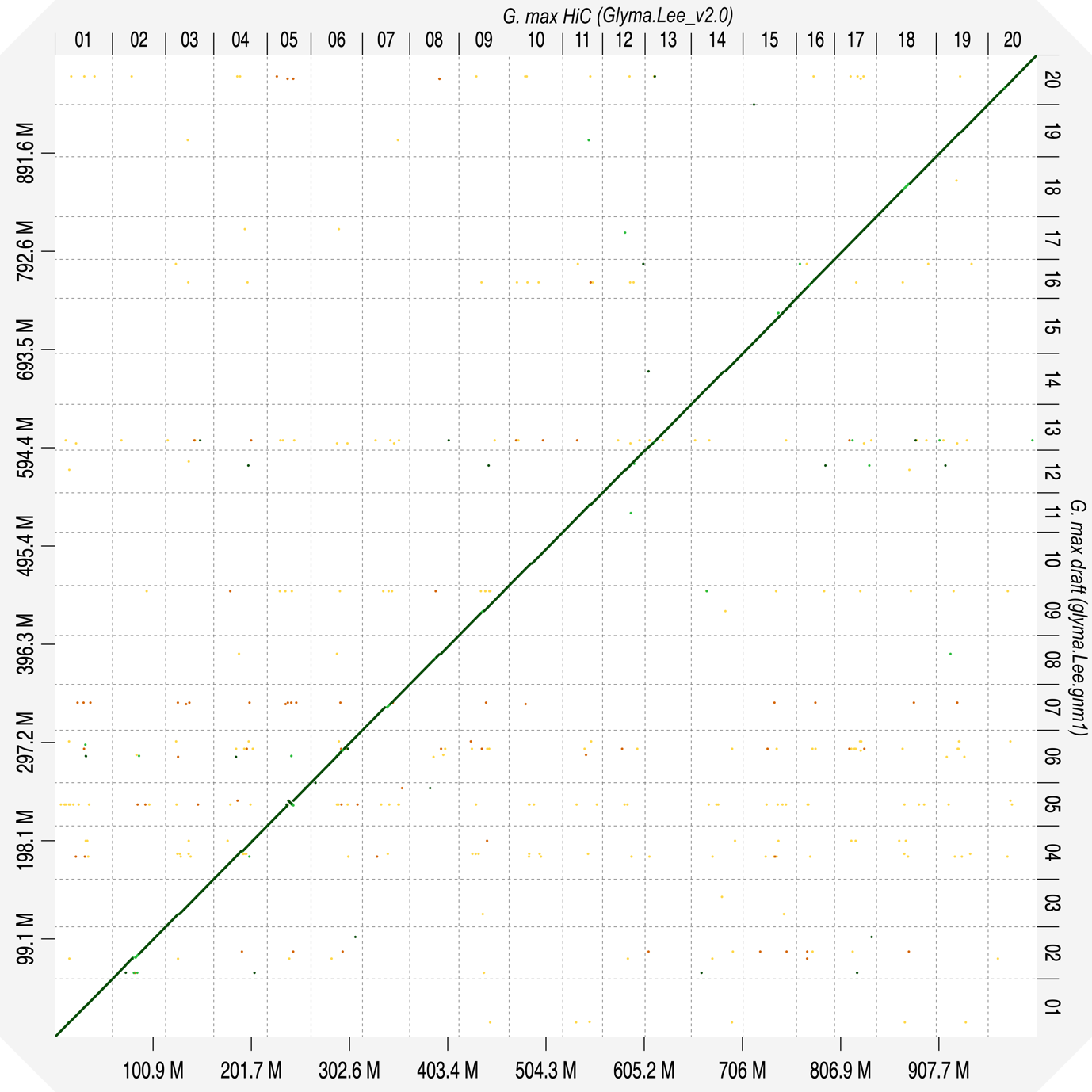


**Fig. S9. Dot plot showing the whole genome alignment between the draft “D” assembly and the Hi-C guided “C” assembly of soybean (*Glycine max*).** The color of the dots represent the percentage identity where green and yellow denote high and low percentage identity, respectively.

**
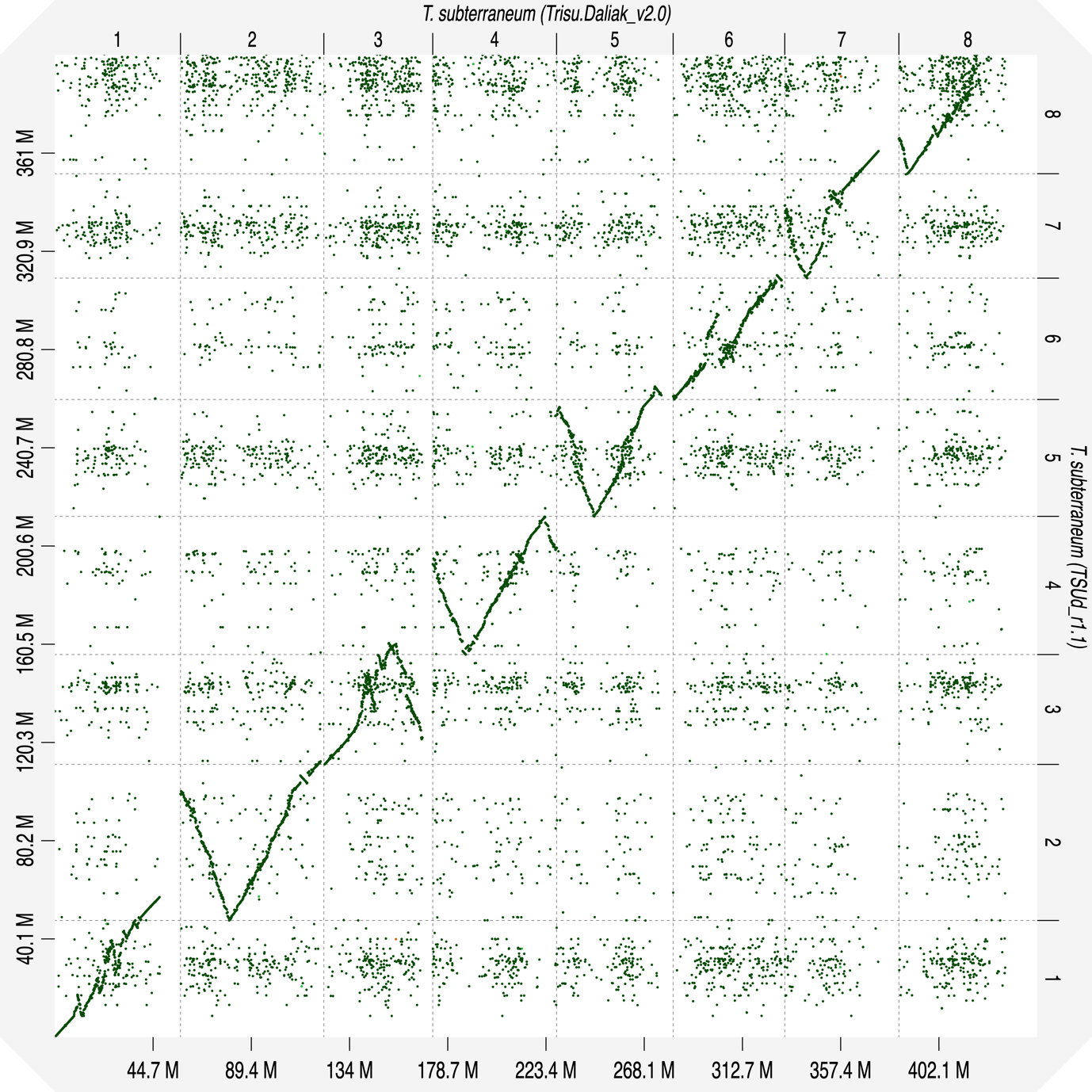
**

**Fig. S10. Dot plot showing the whole genome alignment between the draft “D” assembly and the Hi-C guided “C” assembly of subterranean clover (*Trifolium subterraneum*).** The color of the dots represent the percentage identity where green and yellow denote high and low percentage identity, respectively.

**Fig. S11. Dot plot showing the whole genome alignment between the draft “D” assembly and the Hi-C guided “C” assembly of *Arachis duranensis*.** The color of the dots represent the percentage identity where green and yellow denote high and low percentage identity, respectively.

**Fig. S12. Dot plot showing the whole genome alignment between the draft “D” assembly and the Hi-C guided “C” assembly of *Arachis ipaensis*.** The color of the dots represent the percentage identity where green and yellow denote high and low percentage identity, respectively.

**Fig. S13. A high-density linkage map of pigeonpea (*Cajanus cajan*) developed using 6868 markers identified from the F_2_ population from ICPA 2039 x ICPL 87119.** The scale on the left side indicates map distance in centimorgans (cM)**.** The eleven linkage groups are shown as vertical bars and each horizontal line on the bars represents a single SNP marker.


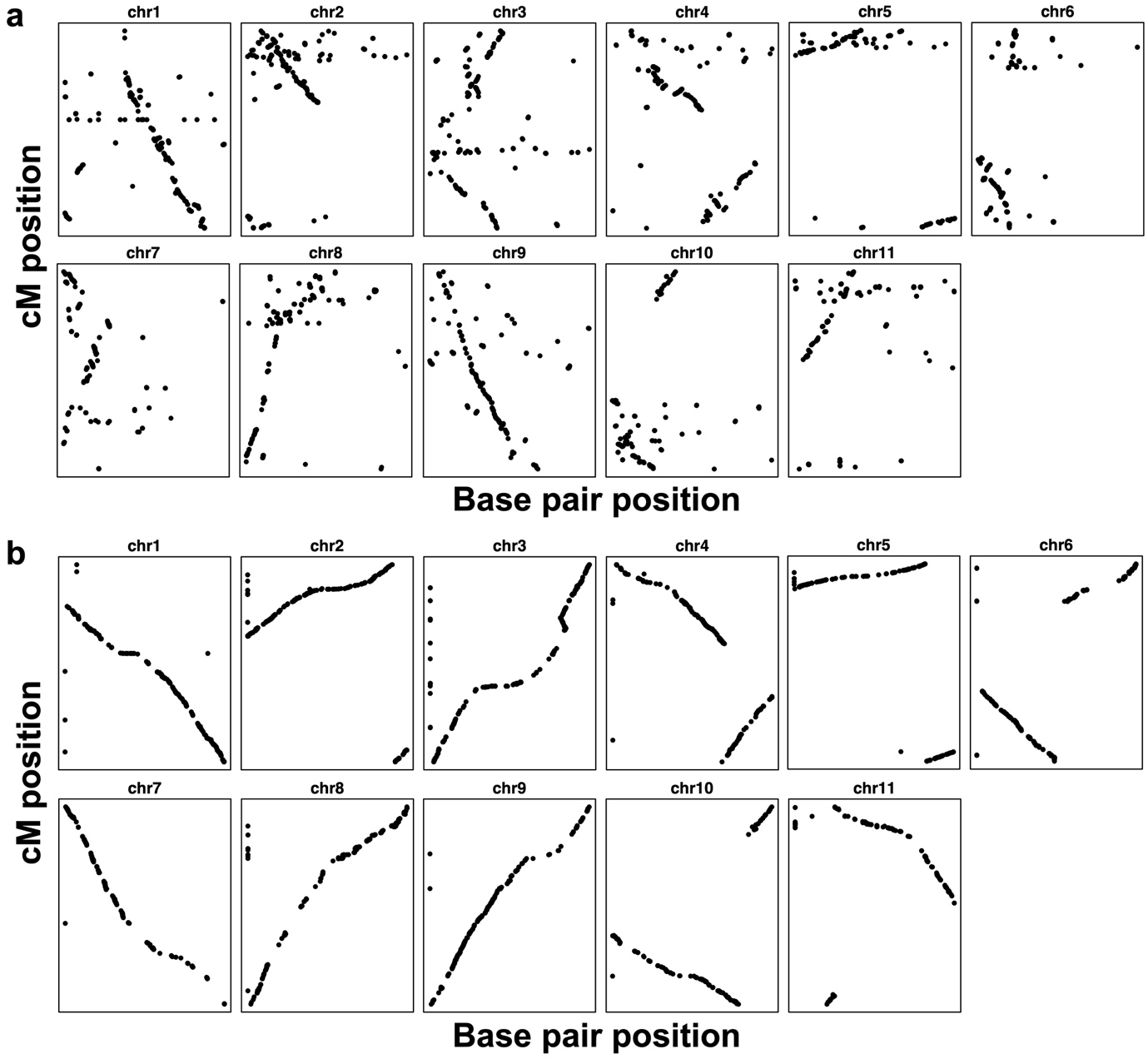


**Fig. S14. Comparison of genetic map and pseudomolecules of pigeonpea (*Cajanus cajan*) (a) draft “D” assembly and (b) Hi-C guided “C” assembly.** The x-axis and y-axis denote the base pair position in the genome assembly and the coordinates of genetic maps, respectively.

**Fig. S15. Homologous dot plot between grape (*Vitis vinifera*) and *Medicago truncatula* genomes.** The best, secondary, and other matched homologous gene pairs were shown by red, blue, and gray dots, respectively. Mean Ks of each inferred colinear block is shown besides. The Ks values <= 1.20 are in red, and others in black, often showing orthology and outparalogy, respectively.

**Fig. S16. Homologous dot plot between grape (*Vitis vinifera*) and *Arachis duranensis* genomes.** The best, secondary, and other matched homologous gene pairs were shown by red, blue, and gray dots, respectively. Mean Ks of each inferred colinear block is shown besides. The Ks values <= 1.10 are in red, and others in black, often showing orthology and outparalogy, respectively.

**Fig. S17. Homologous dot plot between grape (*Vitis vinifera*) and pigeonpea (*Cajanus cajan*) genomes.** The best, secondary, and other matched homologous gene pairs were shown by red, blue, and gray dots, respectively. Mean Ks of each inferred colinear block is shown besides. The Ks values <= 1.10 are in red, and others in black, often showing orthology and outparalogy, respectively.

**Fig. S18. Homologous dot plot between grape (*Vitis vinifera*) and soybean (*Glycine max*) genomes.** The best, secondary, and other matched homologous gene pairs were shown by red, blue, and gray dots, respectively. Mean Ks of each inferred colinear block is shown besides. The Ks values <= 1.10 are in red, and others in black, often showing orthology and outparalogy, respectively.

**Fig. S19. Homologous dot plot between *Medicago truncatula* and *Arachis duranensis* genomes.** The best, secondary, and other matched homologous gene pairs were shown by red, blue, and gray dots, respectively. Mean Ks of each inferred colinear block is shown besides. The Ks values <= 0.80 are in red, and others in black, often showing orthology and outparalogy, respectively.

**Fig. S20. Homologous dot plot between *Medicago truncatula* and soybean (*Glycine max*) genomes.** The best, secondary, and other matched homologous gene pairs were shown by red, blue, and gray dots, respectively. Mean Ks of each inferred colinear block is shown besides. The Ks values <= 0.60 are in red, and others in black, often showing orthology and outparalogy, respectively.

**Fig. S21. Homologous dot plot between *Medicago truncatula* and subterranean clover (*Trifolium subterraneum*) genomes.** The best, secondary, and other matched homologous gene pairs were shown by red, blue, and gray dots, respectively. Mean Ks of each inferred colinear block is shown besides. The Ks values <= 0.60 are in red, and others in black, often showing orthology and outparalogy, respectively.

**Fig. S22. Homologous dot plot between pigeonpea (*Cajanus cajan*) and subterranean clover (*Trifolium subterraneum*) genomes.** The best, secondary, and other matched homologous gene pairs were shown by red, blue, and gray dots, respectively. Mean Ks of each inferred colinear block is shown besides. The Ks values <= 0.60 are in red, and others in black, often showing orthology and outparalogy, respectively.

**
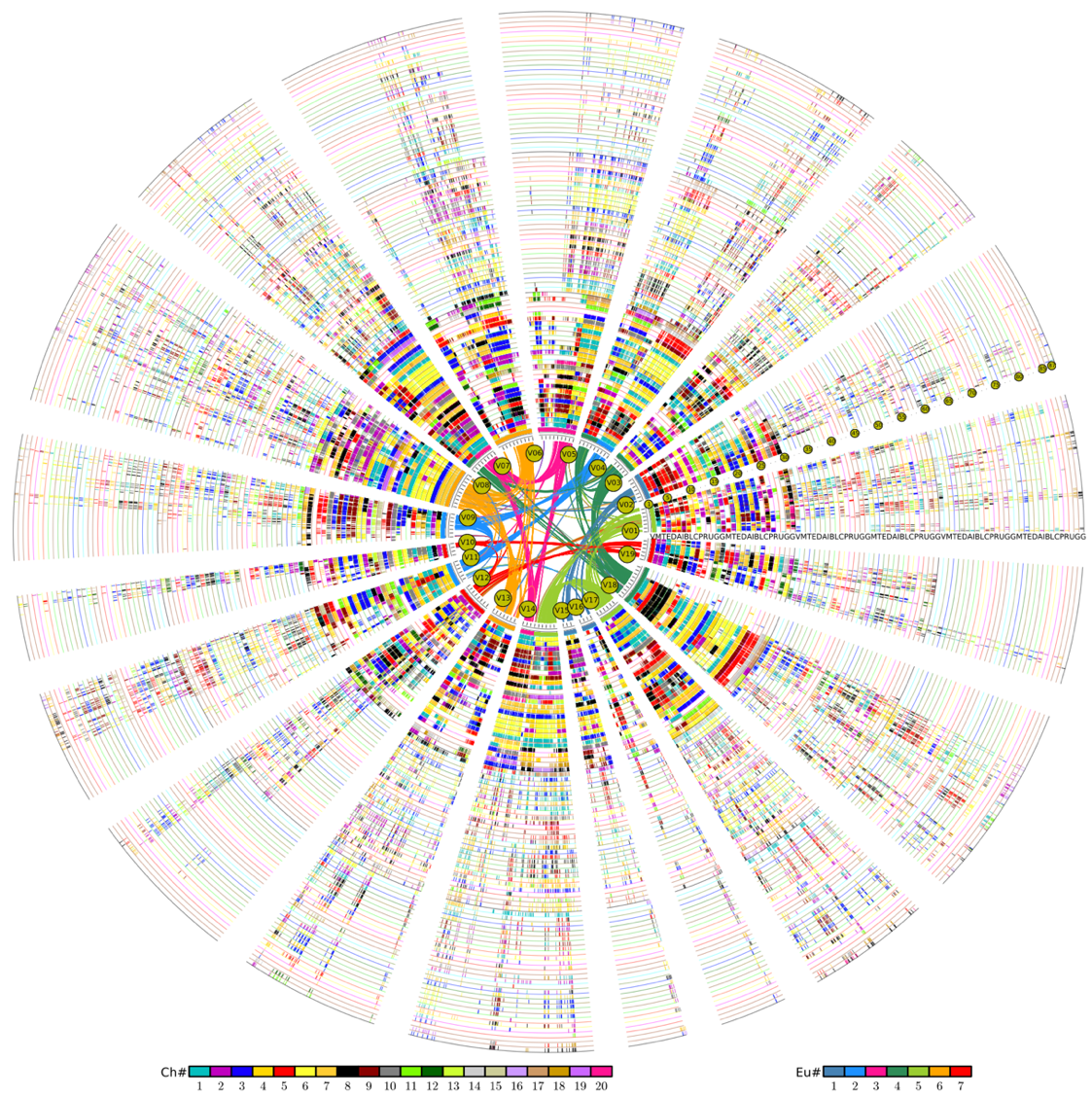
**

**Fig. S23. Homologous alignments of legume genomes with grape (*Vitis vinifera*) as a reference.** Genomic paralogy, orthology, and outparalogy information within and among 12 legumes, are displayed in 87 circles, each corresponding to an extant gene and with name abbreviations (V, *Vitis vinifera*; M, *Medicago truncatula*; T, *Trifolium subterraneum*; E, *Cicer arietinum*; D, *Arachis duranensis*; A, *Arachis hypogaea* A-subgenome; I, *Arachis ipaensis*; B, *A. hypogaea* B-subgenome; L, *Lotus japonicus*; C, *Cajanus cajan*; P, *Phaseolus vulgaris*; R, *Vigna radiata*; U, *Vigna angularis*; G, *Glycine max*). The curved lines within the inner circle are formed by 19 grape chromosomes color coded to correspond to the seven ancestral chromosomes before the ECH. The short lines forming the innermost grape chromosome circles represent predicted genes, which have two sets of paralogous regions, forming another two circles. Each of the three sets of grape paralogous chromosomal regions has two orthologous copies in a legume, with the exception of soybean, which has four. The resulting 87 circles are marked according to species by a capital letter. Each circle is formed by short vertical lines that denote homologous genes, colored to indicate chromosome number in their respective source plant as shown in the color scheme at bottom.

**
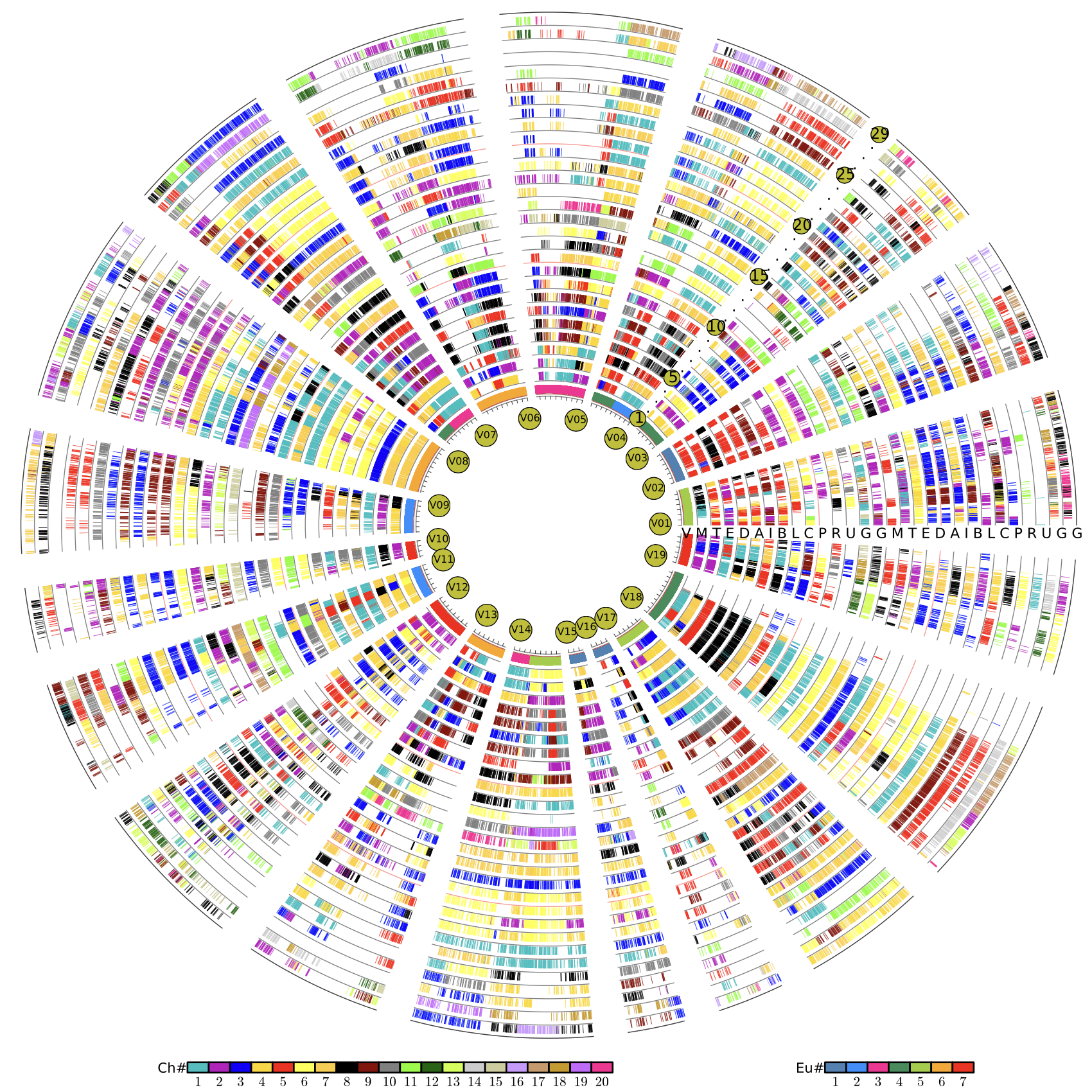
**

**Fig. S24. Orthologous alignments of legume genomes with grape (*Vitis vinifera*) as a reference.** Genomic orthology information as to grape chromosomes and genes within and among 12 legumes are displayed in 29 circles, each corresponding to an extant gene and with name abbreviations (V, *Vitis vinifera*; M, *Medicago truncatula*; T, *Trifolium subterraneum*; E, *Cicer arietinum*; D, *Arachis duranensis*; A, *Arachis hypogaea* A-subgenome; I, *Arachis ipaensis*; B, *A. hypogaea* B-subgenome; L, *Lotus japonicus*; C, *Cajanus cajan*; P, *Phaseolus vulgaris*; R, *Vigna radiata*; U, *Vigna angularis*; G, *Glycine max*). Each of the two sets of grape paralogous chromosomal regions have one orthologous copy in a legume with exception of soybean, which would have 2. Cultivated groundnut subgenomes (A and B) were considered as two different species. Therefore, 12 legumes and one reference genome (grape) resulted in 29 (12x2 + 4 + 1) circles in the figure. The resulting 29 circles are marked according to species by a capital letter. Each circle is formed by short vertical lines that denote homologous genes, colored to indicate chromosome number in their respective source plant, as shown in the color scheme at the bottom.

**
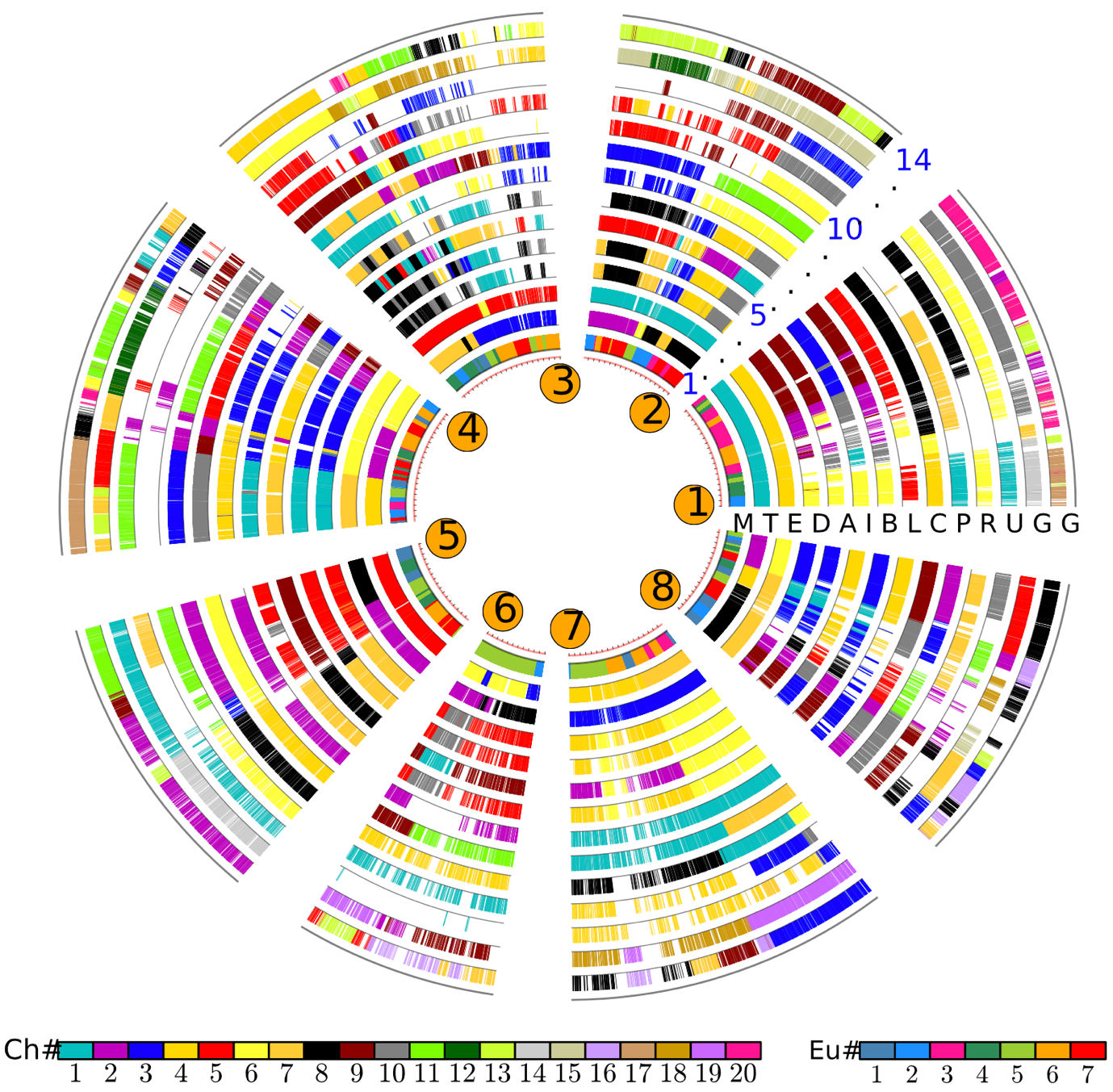
**

**Fig. S25. Homologous alignments of legume genomes with *Medicago truncatula* as a reference.** Genomic orthology, and outparalogy information within and among 12 legumes, are displayed in 14 circles. The resulting 14 circles are marked according to species by a capital letter (M, *Medicago truncatula*; T, *Trifolium subterraneum*; E, *Cicer arietinum*; D, *Arachis duranensis*; A, *Arachis hypogaea* A-subgenome; I, *Arachis ipaensis*; B, *A. hypogaea* B-subgenome; L, *Lotus japonicus*; C, *Cajanus cajan*; P, *Phaseolus vulgaris*; R, *Vigna radiata*; U, *Vigna angularis*; G, *Glycine max*). Each circle is formed by short vertical lines that denote homologous genes, colored to indicate chromosome number in their respective source plant as shown in the color scheme at bottom.


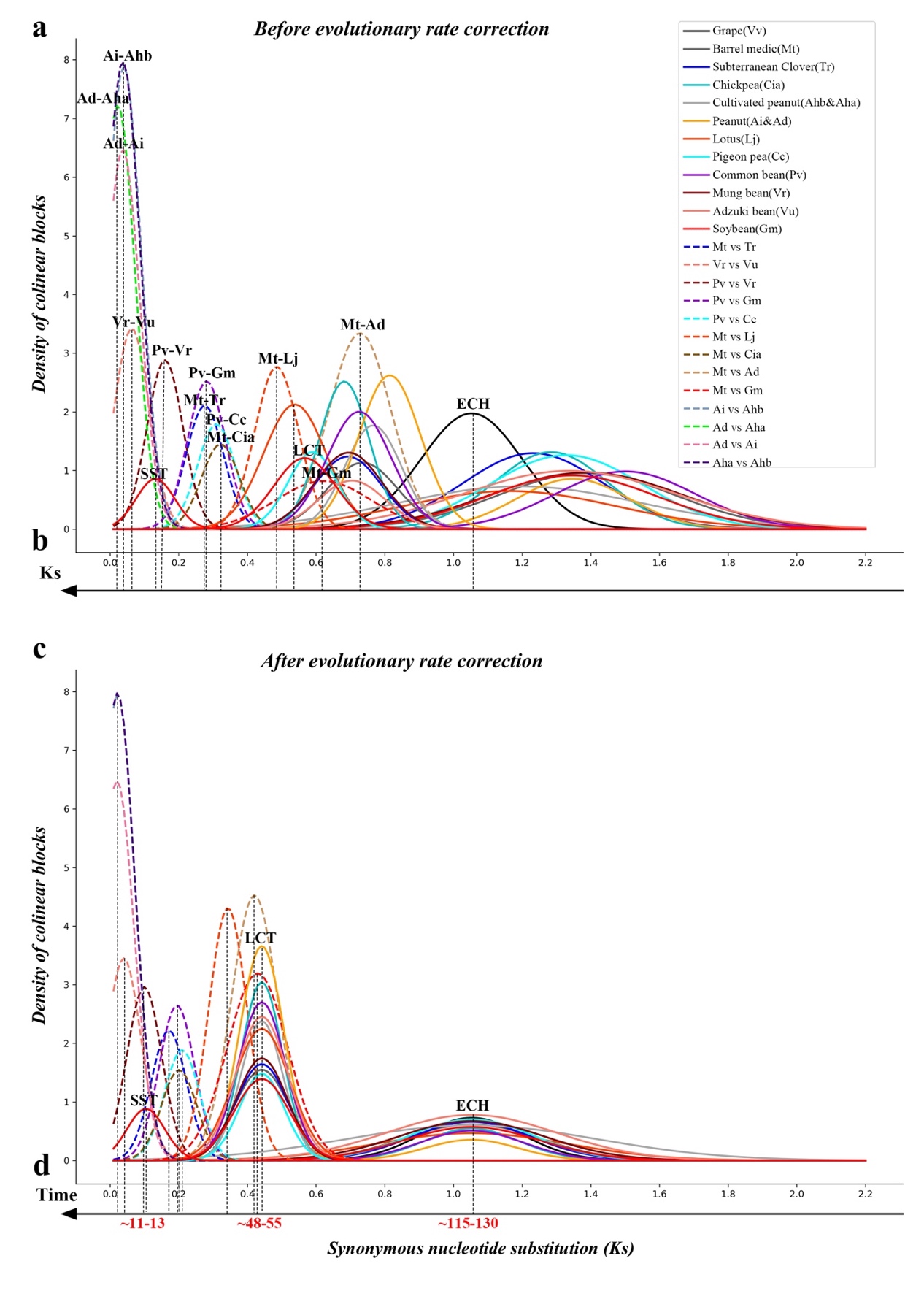


**Fig. S26. Dating evolutionary events within and among the legume genomes.** (a) Distribution of average Ks levels between colinear gene pairs in intergenomic (solid curves) and intragenomic blocks (dashed curves). (b) Ks distribution. (c) Distribution of average Ks levels after correction to account for the evolutionary rate of soybean genes. (d) Correction to the Ks distribution and occurrence of key evolutionary events.

**Fig. S27. Distribution of NBS encoding genes in chickpea (*Cicer arietinum*).** The color scale represents total gene density along the pseudomolecules. The red triangles indicate the location of NBS genes on the pseudomolecules.

**Fig. S28. Distribution of NBS encoding genes in pigeonpea (*Cajanus cajan*).** The color scale represents total gene density along the pseudomolecules. The red triangles indicate the location of NBS genes on the pseudomolecules.

**Fig. S29. Distribution of NBS encoding genes in subterranean clover (*Trifolium subterraneum*).** The color scale represents total gene density along the pseudomolecules. The red triangles indicate the location of NBS genes on the pseudomolecules.

**Fig. S30. Distribution of NBS encoding genes in soybean (*Glycine max*).** The color scale represents total gene density along the pseudomolecules. The red triangles indicate the location of NBS genes on the pseudomolecules.

**Fig. S31. Distribution of NBS encoding genes in *Arachis duranensis*.** The color scale represents total gene density along the pseudomolecules. The red triangles indicate the location of NBS genes on the pseudomolecules.

**Fig. S32. Distribution of NBS encoding genes in *Arachis ipaensis*.** The color scale represents total gene density along the pseudomolecules. The red triangles indicate the location of NBS genes on the pseudomolecules.

**Fig. S33. Distribution of transcription factor encoding genes in the studied legumes.** The stacked barplots depict the number of genes encoding for the most abundant transcription factor families in each legume.

**Fig. S34. Recombination breakpoints identified in 231 chickpea (*Cicer arietinum*) recombinant inbred lines (RILs).** A total of 85,598 identified SNPs were scored as “A” and “B” representing alleles from the two parents ICC 4958 and ICC 1882, respectively. The sliding window (15 bp) based bin mapping approach was used to identify true recombination breakpoints and 2495 bins were identified. Red and green bars represent segments from ICC 4958 and ICC 1882 genotypes, respectively. The black and white panel at the bottom indicates the consensus 2495 bins identified in the entire RIL population.


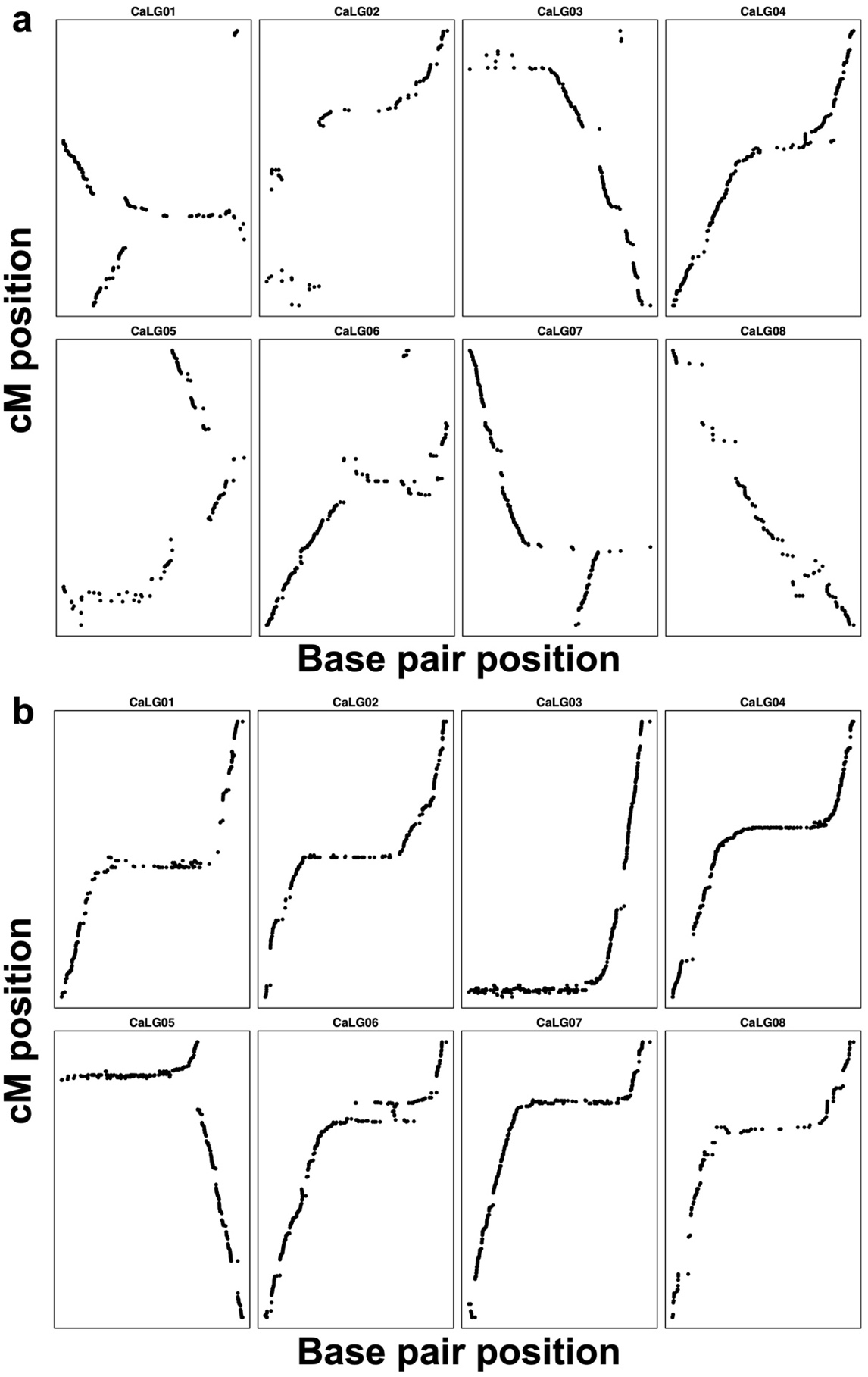


**Fig. S35. Comparison of genetic map and pseudomolecules of chickpea (*Cicer arietinum*) (a) draft “D” assembly and (b) Hi-C guided “C” assembly.** The x-axis and y-axis denote the base pair position in the genome assembly and the coordinates of genetic maps, respectively.
